# Tumor buster - where will the CAR-T cell therapy ‘missile’ go?

**DOI:** 10.1186/s12943-022-01669-8

**Published:** 2022-10-19

**Authors:** Chunrun Qu, Hao Zhang, Hui Cao, Lanhua Tang, Haoyang Mo, Fangkun Liu, Liyang Zhang, Zhenjie Yi, Lifu Long, Luzhe Yan, Zeyu Wang, Nan Zhang, Peng Luo, Jian Zhang, Zaoqu Liu, Weijie Ye, Zhixiong Liu, Quan Cheng

**Affiliations:** 1 Department of Neurosurgery, Xiangya Hospital, Central South University, Changsha, Hunan China; 2 XiangYa School of Medicine, Central South University, Changsha, Hunan China; 3 National Clinical Research Center for Geriatric Disorders, Xiangya Hospital, Central South University, Changsha, Hunan China; 4 Department of Neurosurgery, The Second Affiliated Hospital, Chongqing Medical University, Chongqing, China; 5 Department of Psychiatry, The Second People’s Hospital of Hunan Province, The Hospital of Hunan University of Chinese Medicine, Changsha, Hunan China; 6 The School of Clinical Medicine, Hunan University of Chinese Medicine, Changsha, Hunan China; 7 Department of Oncology, Xiangya Hospital, Central South University, Changsha, Hunan China; 8 One-third Lab, College of Bioinformatics Science and Technology, Harbin Medical University, Harbin, Heilongjiang China; 9 Department of Oncology, Zhujiang Hospital, Southern Medical University, Guangzhou, Guangdong China; 10 Department of Interventional Radiology, The First Affiliated Hospital of Zhengzhou, Zhengzhou, Henan China; 11 Department of Clinical Pharmacology, Xiangya Hospital, Central South University, Changsha, Hunan China

**Keywords:** CAR-T cell, Immunotherapy, Target, Cancer, Personalized treatment

## Abstract

Chimeric antigen receptor (CAR) T cell (CAR-T cell) therapy based on gene editing technology represents a significant breakthrough in personalized immunotherapy for human cancer. This strategy uses genetic modification to enable T cells to target tumor-specific antigens, attack specific cancer cells, and bypass tumor cell apoptosis avoidance mechanisms to some extent. This method has been extensively used to treat hematologic diseases, but the therapeutic effect in solid tumors is not ideal. Tumor antigen escape, treatment-related toxicity, and the immunosuppressive tumor microenvironment (TME) limit their use of it. Target selection is the most critical aspect in determining the prognosis of patients receiving this treatment. This review provides a comprehensive summary of all therapeutic targets used in the clinic or shown promising potential. We summarize CAR-T cell therapies’ clinical trials, applications, research frontiers, and limitations in treating different cancers. We also explore coping strategies when encountering sub-optimal tumor-associated antigens (TAA) or TAA loss. Moreover, the importance of CAR-T cell therapy in cancer immunotherapy is emphasized.

## Background

CAR-T cell therapy specific to tumor antigens is a rapidly evolving concept that has shown remarkable results when applied clinically and has transformed the treatment paradigm for hematologic malignancies. In August of 2017, the Food and Drug Administration (FDA) of the United States approved the use of CAR-T cell therapy in treating patients who suffered from relapsed or refractory B-acute lymphoblastic leukemia (r/r B-ALL). Since then, this field has entered an era of fast-paced, innovative development. Many clinical trials of CAR-T cell therapy have been conducted over the years.

It is widely acknowledged that each T cell has an extremely sensitive and specific T cell receptor (TCR) that constantly checks the organism for ‘non-self’ signals and triggers a cascade of immune responses when abnormal peptides are identified as a precise killer of pathogens. In the TME, T cells are specific to the mutant proteins of cancer cells. Interestingly, these cells could be extracted from a patient’s tumor tissue. After amplification in vitro, they are injected back into the patient, producing a long-lasting antitumor response. However, the method is mainly used for solid tumor treatment and is limited by the collection method, amplification effect, etc. The production scale is small, and its application in the clinic is not satisfactory [1]. CAR-T cell therapy involves genetically engineering T cells to express antigen-specific, non-MHC-restricted receptors that could target and attack specific pathological cells to exert a therapeutic effect on patients [2, 3].

**Fig. 1:**
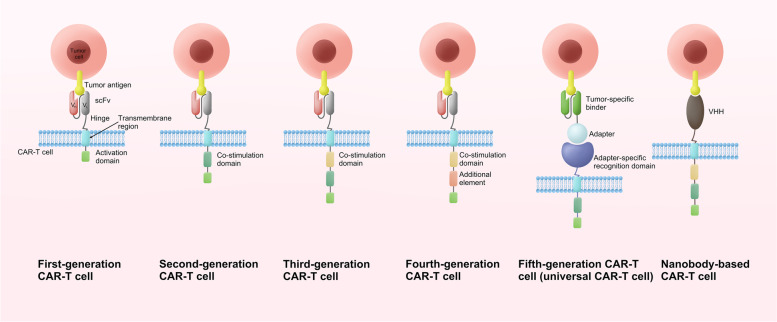
The evolution of CAR-T cell structure. There are five generations of CAR-T cells to date. scFv is currently the most commonly used targeting domain for CAR-T cells, and VHH is also emerging as a targeting domain for CAR-T cells with great potential. V_H_: heavy chain variable domain; V_L_: light chain variable domain; scFv: single-chain fragment variable; VHH: single variable domain on a heavy chain

The structure of CAR has been constantly updated (Fig. 1). First-generation CAR consisted of an extracellular structural domain recognizing antigen and a single intracellular motif. Still, there were no costimulatory molecules in the structure, making it difficult for CAR-T cells to persist in patients and ineffective against tumours [4]. Second-generation CARs have added an intracellular motif consisting of the signaling domain of a costimulatory receptor to their structure [5]. Even in the absence of exogenous costimulatory molecules, second-generation CAR-T cells could continue to proliferate and release cytokines to exert anti-tumor effects and are the most widely used in clinical practice [6]. The third generation CAR contains two costimulatory molecules designed to enhance further the killing ability of CAR-T cells [7, 8]. The fourth generation CARs inserted additional molecular elements to express functional transgenic proteins, such as interleukin genes or suicide genes, enhancing the killing power and safety of CAR-T cells [9, 10]. To improve the flexibility in target recognition of the CAR, the universal CAR-T cells are designed using BBIR CAR or SUPRA CAR. The tumour-specific scFv extracellular structural domain used in previous generations of CAR-T cells is replaced in the universal CAR-T cells with an adapter-specific recognition structural domain that binds to an adapter molecule targeting a tumour-specific target. This design allows the antigen-targeting structural domain to be separated from the t-cell signalling unit, thus giving CAR-T cells the ability to recognise multiple antigens. At the same time, this CAR-T cell only functions to recognise and attack cells when the adapter is provided, thus increasing the user’s control over the CAR-T cell and facilitating its use in the body [11–13]. In addition, single-domain antibodies, also known as single variable domain on a heavy chain (VHH) or nano-antibodies, are also used as targeting domains for CAR-T. Nanobody-based CAR-T cells have been proved to inhibit the growth of solid tumors in immunocompetent mice [14]. Moreover, nanobodies could not aggregate on the surface of T cells because of their monomeric structure [15]. Furthermore, nanobodies do not have the limitation of affinity loss which is recognized as a possible side effect in the design of the conventional single-chain fragment variable (scFv) used as the antigen-targeting domain of CAR [16].

The fact that tumor recognition is not dependent on the major histocompatibility complex (MHC) constitutes their primary benefit. Even though CAR-T cell treatment has shown promising outcomes in clinical trials, considerable challenges remain in cancer treatment using CAR-T cells, such as tumor antigen escape and treatment-related toxicity [17]. CAR-T cell therapies for solid tumors face more significant difficulties due to tumor antigen heterogeneity, difficulty transporting to and infiltrating tumor sites, and challenges with immunosuppressive TME.

The selection of optimal target antigens is the key to addressing these challenges. Typically, the target ought to be a protein, carbohydrate, or glycolipid molecule particularly common in cancer cells. The specificity of the target antigen is essential to prevent toxic effects; the ideal target should be minimally expressed in normal tissue. It is well-recognized that cancer cells could evolve through complex genomic evolutionary mechanisms to evade their destruction by immune cells gradually. Consequently, the target antigen’s stability is vital in avoiding the immunological escape of malignancies. For the security and efficacy of CAR-T cells, ideal targets should include high levels of malignant cell coverage, specificity, and stability [18]. Indeed, antigens that play a crucial role in disease pathophysiology are more suitable as targets. Researchers focus on multi-antigen targeted CAR-T cell therapy to prevent relapse following treatment directed toward a single antigen. This review summarizes and discusses CAR-T cell therapies for different targets in hematological diseases and solid tumors, ranked by disease incidence in Western countries, and highlights the importance of CAR-T cells in oncology treatment.

## Haematologic cancers

### Lymphoma

#### B-cell non-Hodgkin’s lymphoma (B-NHL)

Non-Hodgkin’s lymphoma (NHL) is the most prevalent hematologic tumor, with diffuse large B-cell lymphoma (DLBCL), mantle cell lymphoma (MCL), and follicular lymphoma (FL) representing the most common types. The conventional treatment includes radiation therapy, chemotherapy, etc. However, about 20–30% of patients develop tolerance to these treatments [19]. Hematopoietic stem cell (HSC) transplantation (HSCT) is effective, but many patients are not candidates for this treatment and are prone to relapse after treatment [20]. However, the anti-CD20 antibody rituximab could significantly improve the prognosis of B-NHL, the prognosis of patients who are resistant to immunochemotherapy or relapse after HSCT is extremely poor [19]. Greatly, CAR-T cell therapy could potentially enhance the prognosis of B-NHL patients.

**Fig. 2:**
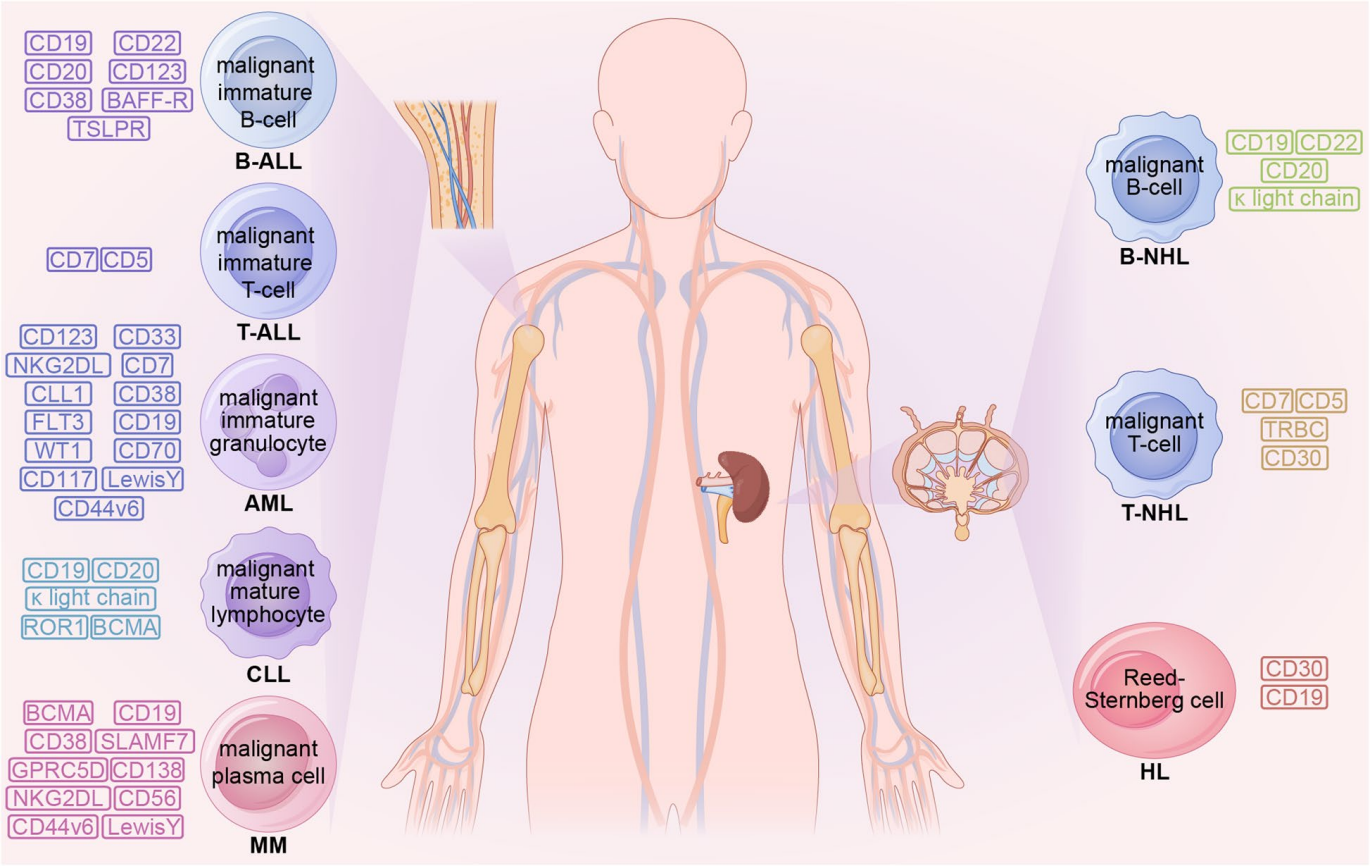
Common CAR-T cell therapy targets in hematological malignancies. B-ALL: B-acute lymphoblastic leukemia; BAFF-R: B-cell activating factor receptor; TSLPR: thymic stromal lymphopoietin receptor; T-ALL: T-acute lymphoblastic leukemia; AML: acute myeloid leukemia; NKG2DL: natural killer group 2 member D ligand; CLL1: C-type lectin like molecule 1; FLT3: FMS-like tyrosine kinase 3; WT1: wilms tumor 1; CLL: chronic lymphocytic leukemia; ROR: receptor tyrosine kinase like orphan receptor; BCMA: B-cell maturation antigen; MM: multiple myeloma; SLAMF7: signaling lymphocytic activation molecule F7; GPRC5D: G protein-coupled receptor class-C group-5 member-D; B-NHL: B-cell non-Hodgkin’s lymphoma; T-NHL: T-cell non-Hodgkin’s lymphoma; TRBC: T cell receptor β-chain constant domains; HL: Hodgkin’s lymphoma

B-NHL is a malignant tumor with high heterogeneity. CD19 is a transmembrane glycoprotein that regulates B lymphocyte activation and proliferation. Its expression in normal tissues is limited to B lymphocyte lines and could be found at high levels in most malignant B cell tumors [21]. The CD19 CAR-T cell therapy product has the highest safety and effectiveness and is the most advanced CAR-T cell therapy product (Fig. 2). Tisagenlecleucel (Kymriah), lisocabtagene maraleucel (Breyanzi), and axicabtagene ciloleucel (Yescarta) are FDA-approved drugs that target CD19 for treating relapsed or refractory DLBCL with good efficacy and manageable adverse events (NCT02445248, JULIET; NCT02631044, TRANSCEND; NCT02348216, ZUMA-1; NCT03391466, ZUMA-7) [22–25]. In a multicenter, single-arm, phase 2 study (ZUMA-12, NCT03761056), axicabtagene ciloleucel yielded highly significant treatment outcomes in 37 patients with high-risk DLBCL, with patients achieving a complete remission (CR) rate (CRR) of 78% and an objective response (OR) rate (ORR) of 89%. Eighty-six percent of patients were alive at the time of data cut-off (median follow-up of 15.9 months), while ≥ grade 3 cytokine release syndrome (CRS) and neurological events occurred in 8 and 23% of patients, respectively. Adverse events were monitored according to the National Cancer Institute’s Common Terminology Criteria for Adverse Events (CTCAE) v5.0 [26]. Compared with the previous median survival of only 6.3 months in high-risk DLBCL patients, CAR-T cell therapy has substantially improved patient survival [19]. The use of axicabtagene ciloleucel is also recommended for treating FL that has recurred or is resistant to therapy [27]. In a multicenter, single-arm, phase 2 trial (ZUMA-5, NCT03105336), 104 patients with relapsed or refractory FL and marginal zone lymphoma were treated with conditioning chemotherapy followed by axicabtagene ciloleucel. Ninety-two percent of patients had an overall response, 74% had a CR. The most common adverse events greater than or equal to grade 3 were haemocytopenia (70% of patients) and infection (18% of patients), which suggests that axicabtagene ciloleucel has good efficacy in indolent non-Hodgkin lymphoma with manageable adverse effects (CTCAE v4.03) [28]. Primary central nervous system (CNS) lymphoma (PCNSL) tends to have a worse prognosis than other lymphomas, and first-line treatment often leads to neurotoxicity. There is little research into treatment options for this disease [29]. In a phase 1/2 clinical trial (NCT02445248), 12 patients with relapsed PCNSL were treated with tisagenlecleucel, of which six patients had a CR, and only one developed immune cell-associated neurotoxicity syndrome, demonstrating the safety and efficacy of tisagenlecleucel in this refractory patient group [30]. Brexucabtagene autoleucel (Tecartus), a CD19 CAR-T cell product, has been given the go-ahead for managing recurrent or refractory MCL. In a phase 2 multicentre clinical trial (ZUMA-2, NCT02601313), 74 patients were enrolled. The primary efficacy analysis showed that 93% of patients receiving brexucabtagene autoleucel had an ORR, 67% had a CR, and estimated progression-free survival (PFS) and overall survival (OS) at 12 months was 61 and 83%, respectively [31]. The everyday adverse events in grade 3 or higher were hematogenic (94%) and infection (32%), with no fatal adverse events (CTCAE v4.03) [31]. In children, Burkitt lymphoma (BL) is perhaps the most prevalent form of NHL [32]. Currently, lentiviral or retroviral technology is often used to produce CAR-T cells. Still, these approaches often hinder CAR expression, carry a high tumor risk, and are more expensive to manufacture [33–37]. A paper published in Nature reports that by using non-viral targeted integration, researchers have prepared CD19 CAR-T cells (AAVS1-19bbz) that effectively eradicate tumor cells in the BL cell line Raji and cell line-derived xenograft mouse models [38]. In this study, the researchers also produced CD19 CAR-T cells (PD1-19bbz) with programmed cell death 1 (PD1) knocked out by CRISPR-Cas9 technology, which showed strong eradication ability against Raji cells that were either high or low in programmed death-ligand 1 (PD-L1) expression [38]. In the phase 1 clinical trial using PD1-19bbz cells, seven out of eight relapsed/refractory B-NHL patients achieved CR and the rest PR, and no CAR-T cell-related grade 3 or higher adverse events were observed (CTCAE v5.0) [38]. This demonstrates the high efficacy and safety profile of the cells. However, there remains a greater likelihood of recurrence after CAR-T cell treatment; the leading cause of relapse is the loss of CD19 molecules. Accordingly, CAR-T cells could be harnessed to target new targets to help solve this problem.

CD22 is a sialic acid adhesin family member which regulates B-cell activation [39] (Fig. 2). It is expressed only in B cell lineages except for plasma cells in normal tissues. It is expressed in most B lymphoma cells and has become a popular therapeutic target for the disease. In a phase 1 dose-escalation study (NCT04088890), three patients had a tumor relapse after recovering from treatment with CD19 CAR-T cells. Still, they had a CR following treatment with CD22 CAR-T cells, and no non-hematological adverse severe events were observed (CTCAE v5.0) [40].

CD20 is highly expressed in malignant cells and could regulate cell activation and proliferation. It has also emerged as an alternative CAR-T therapeutic target (Fig. 2). Clinical trials have confirmed its good efficacy, and no serious adverse effects have been observed in the trials (NCT00621452, 12 participants, CTCAE v3.0; NCT01735604, 50 participants, CTCAE v3.0) [41, 42]. A study reported that a patient with BL with no significant response to CD19 CAR-T cell therapy experienced partial remission (PR) but rapidly relapsed after CD22 CAR-T cell therapy. After CD20 CAR-T cell therapy, he achieved CR with event-free survival (EFS) of 16 months (CTCAE v4.03) [43].

A phase 1 clinical trial (54 participants) of κ light chain CAR-T cell therapy treating B-NHL confirmed that the κ light chain is a prospective target and that this type of CAR-T cell has definite anti-lymphoma activity while ensuring feasibility and safety (NCT00881920, CTCAE v4.03) [44] (Fig. 2).

The most common B-NHL subtype associated with λ light chain expression is MCL, which has an λ:κ expression ratio of approximately 2:1. The efficacy of λ CAR-T has been demonstrated in Igλ + lymphoma cell lines (Maver-1, SP53) and xenograft Igλ + lymphoma mouse models [45].

In addition, chemokine receptor (CXCR) 5 CAR-T cells could target both B-NHL cells and follicular T helper cells, effectively inhibiting lymphoma growth in a mouse xenograft model [46].

Bispecific CAR-T cells are gradually being used in the treatment of B-NHL. Recently, CAR-T cell treatments targeting CD19/CD22 and CD19/CD20 have proven to be highly successful in clinical studies (NCT03233854, NCT03196830, ChiCTR1800015575, NCT03097770, NCT03019055) [47–51]. Such CAR-T cells are particularly helpful in addressing the problem of disease relapse due to antigen loss and deserve the attention of clinicians.

#### T-cell non-Hodgkin’s lymphoma (T-NHL)

Few effective treatments are available for T-NHL, and patients generally have poor prognoses. Moreover, the relapse rate of this disease group is high. Despite CAR-T cell therapy’s relatively good results in treating B-cell malignancies, its application to T-NHL still faces many difficulties. Firstly, manufacturing autologous CAR-T products is difficult because the malignant cells are presented with normal T cells when immune cell extraction is performed on the patient [52]. Secondly, the major CAR-T targeting antigens (e.g., CD5 and CD7) are also expressed in normal T cells [53–55]. The use of CAR-T cells results in the clearance of normal T cells referred to as T cell dysplasia [56]. In addition, target antigens expressed in CAR-T cells themselves could cause CAR-T cells to attack each other, i.e., fratricide [57]. These factors limit the use of CAR-T cell therapy in this disease.

CD5 is a characteristic surface marker of malignant T cells that is extremely important for cell survival and is only expressed in a subset of immune system cells in normal tissues [58, 59] (Fig. 2). The investigators created a CD5 CAR-T cell that could secrete IL-15 with the enhanced anti-tumor response, which rapidly and potently improved the condition of a T-NHL patient with CNS involvement in a phase 1 clinical trial with 20 participants enrolled (NCT04594135) and only grade 1 CRS was observed (CTCAE v4.0) [60]. CD5 is rapidly internalized upon binding to ligands, leading to a reduction in its availability on the cell surface and a consequent decrease in CAR-CD5 interactions [61]. CAR-T cells targeting CD5 downregulate their CD5 expression to counteract self-mutilation and ensure their ability to continue to function [60]. In addition, IL-15 is thought to promote T cell proliferation, which could reduce the impact of self-mutilation on T cell numbers [62]. This minimizes the impact effect of fratricide in this CAR-T cell type. This trial suggests that CD5 CAR-T cells may be an excellent way to treat T-NHL, but further large-scale trials are needed to validate this.

CD7 is expressed in T-NHL malignant cells, normal T cells, and natural killer cells (NK cells). CAR-T cell therapy targeting CD7 is reportedly effective in T-NHL in preclinical studies [53] (Fig. 2).

The mutually exclusive expression of T cell receptor β-chain constant domains (TRBC) 1 and 2 enables immunotherapy to completely eradicate malignant T cells while maintaining a sufficient number of normal T cells to sustain cell-mediated immunity [63]. This could be extremely important for the application of CAR-T therapeutic approaches (Fig. 2).

CD30 CAR-T cells are based on the novel costimulatory combination CD28.OX40 showed vigorous anti-lymphoma activity in anaplastic large cell lymphoma xenograft immunodeficient mouse model, which has an excellent prospect in clinical application [64] (Fig. 2). The costimulatory combination plays a vital role in this.

CD4 is expressed in most T-cell lymphoma subtypes. Patients with peripheral T-cell lymphoma, not otherwise specified, most commonly have a CD4+/CD8- phenotype, with only a few being CD4/CD8 +/+ or −/−, demonstrating the potential of anti-CD4 CAR-T cells [65].

Bispecific CAR-T cells are currently being explored for use in T-NHL. Researchers made CD5/CD7 CAR-T cells that showed anti-tumor substantial effects in malignant T cell lines such as Jurkat, CCRF-CEM, MOLT, and in xenogeneic mouse models established using CCRF-CEM-ffLuc cell injections [66]. The following clinical trials are urgently needed to validate their clinical efficacy and safety.

#### Hodgkin’s lymphoma (HL)

HL is a common type of B-cell lymphoma. First-line therapy is highly effective against these B-cell malignancies. However, more than 10% of patients experience disease progression after initial treatment, with higher relapse rates and limited treatment options for relapsed or refractory HL [67].

It is widely acknowledged that HL malignant cells express CD30 in abundance, and HL, after relapse, still has high CD30 expression. Interestingly, CD30 CAR-T cells could attack tumor cells with low CD30 expression and kill tumor cells that have lost sensitivity to vibutuximab (Fig. 2). In two parallel phases, 1/2 studies conducted at two independent centers (NCT02690545, NCT02917083), the ORR for 32 patients receiving a lymphatic clearance regimen followed by infusion of CD30 CAR-T cells was 72%, with 59% of patients having CR. Grade 3 or higher hematological adverse events were the most common toxicity, and no CRS or neurotoxicity beyond grade 1 (CRS, Lee 2014 criteria; other adverse events, CTCAE v4.0) [68]. The trial also found that 27 r/r HL patients who received CD30 CAR-T cells after a lymphatic clearance had a median PFS of 352 days (NCT02690545) [69]. Despite the high clinical response rate, more extensive clinical trials are needed to verify its clinical effectiveness. Humanized scFv-based CD30 CAR-T cell has low immunogenicity, low risk of cytokine-mediated toxicity, and high persistence. It destroyed CD30+ tumor cell lines (L428 and L540) in vitro and cleared lymphoma in lymphoma-bearing mice, showing promising efficacy for the next phase of clinical trials [70]. The application of the co-stimulation combination CD28.OX40 is of great value in improving the effectiveness of CD30 CAR-T cells and is expected to be used in the following clinical trials [64].

CD19 could also be used as a therapeutic target (Fig. 2). According to research (ChiCTR2000028922), a patient treated with CD19 and CD30 CAR-T cells showed a protracted PFS and no severe adverse effects (CTCAE v4.0) [71].

### Leukemia

#### B-acute lymphoblastic leukemia (B-ALL)

B-ALL is caused by malignant precursor B lymphocytes that affect the production of normal blood cells in the bone marrow. It is much more prevalent in adults than T-acute lymphoblastic leukemia (T-ALL) [72]. Chemotherapy is the current first-line treatment for B-ALL. However, some patients develop relapsed or refractory acute B-cell lymphoblastic leukemia (r/r B-ALL) after conventional chemotherapy and have a poor prognosis. Current evidence suggests that CAR-T cell therapy plays a significant role in treating r/r B-ALL.

CD19 is now the most commonly used and researched CAR-T target for B-ALL treatment (Fig. 2). The first CAR-T cell treatment approved by the FDA to treat r/r B-ALL is tisagenlecleucel targeting CD19. In a phase 2 study conducted in 25 centers (ELIANA, NCT02435849), 75 patients received tisagenlecleucel infusions with an ORR of 81% at 3 months, EFS rate and the rate at 12 months were 50 and 76%, respectively, and a 73% incidence of grade 3 or 4 adverse events possibly related to treatment [73]. In another phase 2 study (ENSIGN, NCT02228096), 20 of 29 patients achieved OR. Eleven had grade 3 or 4 CRS, and one had grade 3 neurological symptoms [74]. Follow-up studies on this group of patients have shown a significant improvement in their quality of life, better than conventional therapy [75]. These two trials have important implications for the commercialization of tisagenlecleucel. The other CAR-T cell product targeting CD19, brexucabtagene autoleucel, is also approved for r/r B-ALL treatment. In a phase 2 clinical trial (ZUMA-3, NCT02614066), 56% of the 55 patients receiving brexucabtagene autoleucel achieved CR, with a median of 18.2 months, and anemia (49%) was the most common adverse event at grade 3 or higher (CRS, Lee 2014 criteria; other adverse events, CTCAE v4.03) [76]. Intravenous immunoglobulin could partially limit the side effects of the attack on normal B cells. Although CD19 CAR-T cell therapy induces very high CRR in B-ALL patients, the recurrence of the disease remains an important issue. The absence or mutation of antigens and the limited duration of CAR-T cell function in vivo may account for relapse after treatment.

In response to disease relapse, researchers have begun constructing CAR-T cells that target different targets and respond by adjusting the manufacturing process. CD22 is expressed in 90% of juvenile and 50–100% of adult patients, suggesting it is an excellent target for relapsed B-ALL treatment [77, 78] (Fig. 2). In a study with CD22 CAR-T cells for r/r B-ALL (ChiCTR-OIC-17013523), 24 of 30 patients achieved CR within 1 month, and the 12-month leukemia-free survival rate for patients was 71.6%, with most patients experiencing only minor adverse effects. No CD22 antigen loss or mutation was found in the limited number of patients who relapsed (CTCAE v4.03) [79].

CD38 has been documented in r/r B-ALL malignant cells, and CD38 CAR-T cells were used to treat an r/r B-ALL patient who failed to respond to bispecific CD19/CD22 CAR-T cell therapy (Fig. 2). However, the patient developed severe complications and abandoned the treatment after 20 days of cell infusion [80].

In addition to the targets mentioned above that have proven their effectiveness in clinical trials, many promising targets are being explored. B-ALL malignant, dendritic, and HSCs express CD123 [81]. CD123 CAR-T cell therapy is an ideal solution for relapse after CD19 CAR-T treatment since it is expressed in most CD19- relapsed or innately CD19- resistant subpopulations (Fig. 2). In animal models, CD123 CAR-T cells have demonstrated high efficacy against CD19- B-ALL cells [82]. However, CD123 is expressed on normal HSCs, so CD123 CAR-T cells could potentially harm the bone marrow.

The B-cell activating factor receptor (BAFF-R) may be retained in recurrent cancer malignant cells (Fig. 2). In numerous xenogeneic animal models, including CD19 antigen deletion models, BAFF-R CAR-T cells could efficiently and accurately remove B-ALL malignant cells [83].

In addition, CRLF2 gene rearrangements produced an r/r B-ALL phenotype insensitive to standard chemotherapy regiments with poor prognosis [84]. Studies have found that thymic stromal lymphopoietin receptor (TSLPR) CAR-T cells soundly affect the subtype of diseases mentioned above [85] (Fig. 2).

The combination of CD19 and CD22 CAR-T cells has attracted significant interest recently (Fig. 2). In a trial of r/r B-ALL patients (ChiCTR-ONC-17013648), serial infusions of CD19 and CD22 CAR-T cells were given to 21 patients who relapsed after HSCT. Twenty patients achieved CR 1 month after the second infusion, including those who relapsed after the first infusion of CD19 CAR-T cells. No grade 3 or higher CRS or neurotoxicity was observed (CRS, Penn grading scale; other adverse events, CTCAE v5.0). The 12-month EFS and rates of patients were 67.5 and 88.5%, respectively. In contrast, 50–57% of patients in the group given only a single CD19 or CD22 CAR-T cells treatment relapsed within 6–8 months [86]. In another trial (NCT03185494), infusion of bispecific CD19/CD22 CAR-T cells to patients with r/r B-ALL resulted in CR in all six patients without severe adverse events (Neurological toxicities, Lee 2014 criteria; CRS, American Society for Transplantation and Cellular Therapy criteria) [87]. In a phase 1 trial conducted in Beijing (ChiCTR-OPN-16008526), 23 r/r B-ALL patients were treated with dual-targeted CD19/CD22 CAR-T cells. All 22 patients are willing to be evaluated for achieved CR, with estimated 12-month PFS rates and rates of 59.2 and 67.4%, respectively. Adverse reactions in patients greater than or equal to grade 3 included haemocytopenia, fever, and CRS. The rest of the adverse reactions were mild (CRS, American Society for Transplantation and Cellular Therapy criteria; other adverse events, CTCAE v4.03) [88]. In addition, one study found that CD19 CAR-T was an independent risk factor associated with severe CRS (ChiCTR1800015575) [89]. Bispecific CD19/CD22 CAR-T cells may lower the risk of CRS [89]. Recently, researchers have created CAR-T cells that target CD19/20/22 by co-expressing a CAR-T cell molecule on T cells using a tricistronic transgene. CD19/20/22 CAR-T cells showed superior cytotoxicity to CD19 CAR-T cells in in vitro assays against Daoy cells and primary B-ALL malignant cells. In the NSG xenograft model, CD19/20/22 CAR-T cells showed more potent inhibition of CD19(−) leukemia cells in patients who failed CD19 CAR-T cell therapy, which was challenging to inhibit CD19 CAR-T cells [90]. Dual/multi-targeted CAR-T cells could improve CRR and even reduce adverse reactions and are promising in clinical applications.

Besides, CD72 was revealed as a target for in vitro-evolved nanobody-based CAR-T cells in KMT2A/MLL1-rearranged B-ALL [91].

#### T-acute lymphoblastic leukemia

Although CAR-T cell therapy has improved the prognosis of r/r B-ALL patients, it has had less impact on T-ALL patients. Similar to what CAR-T cells face in T-NHL treatment, normal and malignant T cells often co-express target antigens, causing CAR-T cells to target normal T cells and causing severe T cell immunodeficiency. As a result, the development of CAR-T cell treatment for T-ALL remains difficult.

CD7 is expressed in 95% of T-ALL malignant cells. Eighteen of 20 T-ALL patients treated with allogeneic CD7 redirected CAR-T cells achieved CR in a single-center phase 1 clinical trial (Fig. 2). In comparison (NCT04689659), grade 3–4 haemocytopenia occurred in all patients, and grade 3–4 CRS occurred in 2 patients (haemocytopenia, CTCAE v5.0; CRS, American Society for Transplantation and Cellular Therapy criteria) [92]. In two trials (ChiCTR190002531, ISRCTN19144142), one of the two patients receiving CD7 CAR-T cells was in sustained remission for more than 1 year after treatment patients experienced grade 3 CRS (American Society for Transplantation and Cellular Therapy criteria) [93]. Despite the excellent effectiveness, the safety of the treatment needs further improvement. An important area of current research is cytosine base editors (CBEs). Unlike the induced DNA double-strand breaks (DSBs) technique used in the manufacture of most allogeneic CAR-T cells, CBEs create point mutations in T cells that silence gene expression without DSBs with an efficiency of 90 to 99%, significantly reducing the incidence of unexpected target editing results [94–96]. Allogeneic CD7 CAR-T cells developed based on CBEs are highly effective against T-ALL cells in a CD7+ T-ALL cell line CCRF-CEM, a model constructed by transplanting CCRF-GFP-Luc cells in NSG mice, and a mouse model created from patient-derived xenografts [96]. In addition, the removal of CD7 expression on the surface of T cells by gene editing technology could significantly inhibit the fratricide of CAR-T cells and reduce the risk of side effects. CD7 and TCR alpha chain-deficient CD7 CAR-T cells (UCART7) manufactured by CRISPR/Cas9 gene editing technology were used in the CD7+ T-ALL cell lines MOLT-3 (ACC 84), MOLT-4 (ACC 362), HSB-2 (ACC 435) and CCRF-CEM (ACC 240), the CCRF-CEM xenograft models and patient-derived xenograft models all showed better anti-tumor effects and significantly reduced fratricide [57].

CD5 is expressed in 80% of T-ALL cells. In vitro, CAR-T cells targeting CD5 successfully kill malignant T cell lines (CCRF-CEM, MOLT-4, and KARPAS-299) and primary T-ALL parent cells (Fig. 2). It also significantly slowed disease progression in a T-ALL xenograft mice model [97]. T-ALL cell lines and primary T-ALL malignant cells have been found to express natural killer group 2 member D ligand (NKG2DL). In healthy cells, it is rarely expressed. NKG2DL CAR-T cells have been shown to have remarkable in vitro activity against T-ALL cell lines (Jurkat, HPB-ALL, KOPT-K1, DND-41) [98].

#### Acute myeloid leukemia (AML)

The incidence of AML is high among adults, and it is the second most prevalent form of pediatric leukemia. In these patients, HSC proliferates uncontrollably and overproduces immature and functionally abnormal white blood cells [99]. Chemotherapy is a commonly used treatment strategy nowadays but often leads to poor outcomes due to the limitations of the approach, such as toxic effects on healthy tissue. HSCT becomes another option. However, the five-year survival rate for patients with relapsed AML is only about 27% [100, 101]. Given that CAR-T cells may specifically target antigens on leukemic stem cells and progenitor cells, they have enormous application potential. However, since many myeloid antigens are also expressed on healthy HSC, the critical challenge currently limiting the adoption of CAR-T cell therapies in this field is appropriate to target selection.

As genealogy-limiting antigens, CD33 and CD123 are currently the most studied CAR-T cell therapeutic targets (Fig. 2). CD33 and CD123 are expressed in approximately 99 and 78% of AML malignant cells, respectively, and CAR-T cell therapies that target them are effective in preclinical trials [102–106]. Many relevant clinical trials are underway. However, CD123, widely expressed in adult AML, may be less represented in children [104]. In addition, myeloid and hematopoietic progenitor cells express CD33 and CD123, which may hinder their practical application [107, 108].

AML-initiating cells express CD38, while normal human HSC does not. In a phase 1/2 clinical trial (NCT04351022), four out of six AML patients treated with CD38 CAR-T cells achieved CR, the median of 7.9 months, with no severe adverse events (CTCAE v4.0) [109] (Fig. 2).

Since it is significantly expressed in AML cells but not in healthy HSC or non-hematological cells, C-type lectin-like molecule 1 (CLL1) represents a promising target for CAR-T cells (Fig. 2). In a phase 1/2 clinical trial of CLL1 CAR-T cells for AML, 3/4 of patients achieved CR, with no high-level adverse events observed (CTCAE v5.0) [110]. Two AML patients who did not recover after multiple lines of salvage therapy, including CD38 CAR-T cell therapy, achieved molecular CR treated with CLL1 CAR-T cell therapy (NCT04884984). And again, there were no high-grade adverse events in patients (CTCAE v5.0) [111]. These trials suggest the great potential of CLL1 CAR-T cells.

LewisY is less expressed in healthy tissues and may also be a good target for AML therapy, given its expression on malignant cells (Fig. 2). In a phase 1 clinical trial (CTX 08–0002), five patients with relapsed AML were enrolled. It was established that the use of LewisY CAR-T cells for the management of AML is feasible and secure [112]. Grade 3 or 4 toxicity was not observed (CTCAE v3.0). One patient achieved cytogenetic remission, one had a reduction in peripheral blood blasts, and one showed prolonged remission.

There are also many promising targets whose effects are not yet supported by clinical trial results. For example, CD7 is expressed by leukemic cells such as AML, which accounts for 30% of all cases, but not normal bone marrow cells (Fig. 2). As a consequence of this, it has the potential to be an intriguing candidate for the selective destruction of cancer cells without affecting the health of normal cells. A study found that CD7 CAR-T cells could effectively eradicate CD7+ AML cell lines (GDM-1 and Kasumi-3), primary CD7+ AML, and colony-forming cells in a xenograft mice model but did not affect the normal cells in bone marrow [113]. Besides, nanobody-based fratricide-resistant CD7-CAR T cells demonstrated a favorable and durable antitumor response in r/r T-ALL/LBL with tolerable toxicity, warranting further studies in highly aggressive CD7-positive malignancies [114]. However, since T cells express CD7, T cell self-mutilation is an issue to be considered for CD7 CAR-T cell therapy.

With standard treatment, the prognosis and clinical outcomes of AML patients with FMS-like tyrosine kinase 3 (FLT3) are poor. Targeted therapy is thus highly anticipated. In a mouse model of AML, FLT3 CAR-T cells allowed for bone marrow recovery without affecting leukemic remission [115] (Fig. 2).

Overwhelming evidence substantiates that CAR-T cells targeting CD117, Siglec-6, CD70, myeloproliferative leukemia protein (MPL), leukocyte immunoglobulin-like receptor-B4 (LILRB4), T cell immunoglobulin and mucin structural domain 3 (TIM-3), membrane-associated folate receptor β (FRβ) and CD44v6 CAR-T cells induce complete remission in immunodeficient mouse xenograft AML models [116–123] (Fig. 2).

Wilms tumor 1 (WT1) overexpression on tumor cells is linked to a poor prognosis in AML patients. In an in vitro assay, WT1 CAR-T cells identified and lyzed WT1+/HLA-A*02:01+ tumor cell lines (AML, AML-14; CML, BV173; ovarian cancer, OVCAR3) [124] (Fig. 2).

CAR-T cells targeting PR1 exhibit a significant affinity in vitro for PR1+ target cells, and they targeted human primary AML cells with a preference [125].

In addition, mesothelin (MSLN) is a possible target of CAR-T cells [126].

#### Chronic lymphocytic leukemia (CLL)

In Western nations, CLL is the most frequent form of adult leukemia, and its onset is associated with advancing age. However, treatment options are limited, the most effective treatment option is HSCT, but it is rarely used due to the high risks. CAR-T cell therapy might also be helpful for individuals with high-risk CLL who have not seen improvement from standard treatment.

CD19 is the main target of CAR-T cells treating CLL patients (Fig. 2). However, CD19 CAR-T is not as effective in CLL as in ALL. Only 45% of 22 CLL patients treated with CD19 CAR-T cells achieved CR in a phase 1 multicenter clinical trial in 2022 (NCT03331198). 74% of patients had CRS (9% grade 3) and 39% had neurological symptoms (22% grade 3 or 4) (CRS, Lee 2014 criteria; other adverse events, CTCAE v4.03) [127]. In a previous study (NCT02640209), CD19 CAR-T treated 14 patients who had a CRR of only 28%. Six patients had grade 3 or higher CRS, one had grade 4 neurological symptoms lasting 2 days, and B cells were undetectable in all CR patients (CRS, American Society for Transplantation and Cellular Therapy criteria; other adverse events, CTCAE v4.03) [128]. Antigen-negative tumor escape also has a high probability of causing recurrence [129, 130]. These findings emphasize the need for new therapeutic targets and improved technologies.

B-cell maturation antigen (BCMA) is found on plasma cells and advanced B lymphocytes. It has been found to have more significant potential for immunotherapy in CLL patients [131] (Fig. 2). Because soluble BCMA levels are negatively linked with time to treatment failure and OS, but not with the CLL International Prognostic Index, therapeutic methods targeting BCMA may improve the prognosis of CLL patients [132].

On the tumor cells of CLL patients, CD32b is always produced at a considerable locus density, but this is not the case in non-B hematopoietic cells. CD32b CAR-T cells showed intense activity in both primary CLL cells and NSG mice transplanted with patient samples [133].

In a preclinical study, FcμR-specific CAR-T cells have successfully eliminated Mec-1 leukemic cells without affecting healthy B cells [134].

Receptor tyrosine kinase like orphan receptor (ROR) 1 is stably expressed in CLL patients and not on normal, healthy differentiated tissue [135]. ROR1 CAR-T cells are particular and could reduce side effects associated with treatment, such as B cell depletion and hypogammaglobulinemia. Therefore, it is an attractive target for CAR-T cell therapy (Fig. 2).

CAR-T cells targeting Siglec-6 and CD23 separately have been developed, and their effects will be confirmed in future experiments [136, 137].

Additionally, kappa and lambda chains are potential targets [138].

The application of bispecific CAR-T cells offers new hope for CLL treatment. In a phase 1 trial (NCT03019055), of 22 patients receiving CD19 and CD20-targeted CAR-T cells, 14 (64%) achieved CR, one (5%) developed grade 3–4 CRS, and three (14%) developed grade 3–4 neurotoxicity, suggesting that this therapeutic approach has good potential (CRS, American Society for Transplantation and Cellular Therapy criteria; other adverse events, CTCAE v5.0) [51].

### Multiple myeloma (MM)

MM is a cancer of the plasma cells, second only to leukemia among hematologic malignancies. Despite substantial advancements over the last two decades, the prognosis for people with MM remains bleak. CAR-T cells have been demonstrated to have potential as a treatment option for patients with recurrent or refractory multiple myeloma (r/r MM).

BCMA is the most effective target for CAR-T cell therapy in MM among the numerous possible targets (Fig. 2). In normal cells, BCMA is primarily expressed by plasma cells and a small percentage of mature B cells, while it is absent from most B cells and other organs. BCMA is a highly desirable target for immunotherapy since it is extensively expressed in MM malignant cells [139]. In 2021, the FDA authorized idecabtagene vicleucel (Abecma) for use in patients with r/r MM who have failed fourth-line therapy. Idecabtagene vicleucel is the first FDA-approved CAR-T cell therapy to manage MM. In a phase 2 study including 128 patients with r/r MM (NCT03361748), patients had an ORR of 73%, a CRR of 33%, and a PFS of 8.8 months, and almost all had grade 3 or 4 toxicities (CRS, Lee 2014 criteria; other adverse events, CTCAE v4.03) [140]. The FDA also approved a second BCMA-targeted CAR-T cell product, ciltacabtagene autoleucel (Carvykti), for the treatment of MM in 2022 [141]. The targeting domain of this CAR-T cell product is based on single-domain antibodies [142]. In a phase 1b/2 trial (CARTITUDE-1, NCT03548207), 67% of the 97 patients who received infusion ciltacabtagene autoleucel achieved CR. The rate at 12 months is 89%. Grade 3 and above hematological adverse events were common in patients, 21% of patients had neurotoxicity, and most patients who experienced CRS remitted, demonstrating the good-excellent efficacy and safety of the product (CRS, American Society for Transplantation and Cellular Therapy criteria; other adverse events, CTCAE v5.0) [142]. In the clinical trial of LCAR-B38M (NCT03090659), 100 participants were enrolled. A nanobody-based BCMA-redirected CAR-T cell treatment (LCAR-B38M) that targets two separate BCMAepitopes showed a 68% CRR, 15 months of PFS, and 65% rate of grade 3 and above adverse events in the patient population, suggesting its good performance (CRS, Lee 2014 criteria; other adverse events, CTCAE v4.03) [143]. An autologous second-generation BCMA-redirected CAR-T constructed on humanized alpaca-derived anti-BCMA nanoantibodies demonstrated safety and efficacy in a trial of 16 patients with r/r MM (NCT03661554). Three patients with extramedullary lesions achieved PR within 1 month, and the overall response rate was 84.6% in the 13 patients without the extramedullary disease. Only two patients had CRS of grade 3 or above; the rest had mild CRS (grade 0 to 2) [144]. A separate report of the results of this clinical trial showed that as of 1 February 2021, 34 patients with MM had received this CAR-T cell with an overall response rate of 88.2% and an mPFS of more than 1 year, with haemocytopenia being the most common adverse effect and all greater than grade 3 (CTCAE v5.0). Twenty nine patients experienced CRS (any grade) (Lee 2014 criteria) [145]. This further confirms the efficacy and safety of nanobody-based BCMA retargeted CAR-T cell therapy for r/r MM patients. In 2021, a meta-analysis counted 22 studies using BCMA CAR-T cells for MM, with mean ORR and CRR of 85.2 and 47.0%, respectively [146]. Recent research found that suppressing elevated anti-apoptotic proteins in MM cells via bone marrow mesenchymal stromal cells could boost the efficiency of BCMA CAR-T cells (ChiCTR1800017051, ChiCTR2000033925) [147]. However, BCMA CAR-T cell therapy is associated with a high prevalence of toxic side effects and recurrence. Combining γ-secretase (GS) inhibitor (GSI) with CAR-T cells targeting BCMA is a possible solution. The GSI inhibited the decrease in antigen concentration caused by GS cleavage of BCMA on the tumor cell surface and the release of soluble BCMA fragments, which could hinder the function of CAR-T cells [148, 149]. In MM tumour-bearing NSG mice treated with GSI, BCMA expression on malignant cells was upregulated, soluble BCMA fragments in peripheral blood were reduced, and the efficacy of BCMA-targeted CAR-T cells was significantly enhanced [150]. Clinical trials combining GSI with CAR-T cells targeting BCMA are already underway (NCT03502577).

Exploring CAR-T cells that target new targets may also be an excellent way to address the problem of relapse. Two out of 10 patients showed significantly longer PFS after HSCT and CD19 CAR-T cell therapy compared with HSCT alone. No patient experienced severe CRS, which demonstrated the potential benefit of CD19 CAR-T cells for r/r MM patients (NCT02135406) [151] (Fig. 2). In a phase 1 trial, seven MM patients received κ light chain CAR-T therapy, four of whom had stable disease for two to seventeen months (NCT00881920). No toxicity attributed to CAR-T cells has been observed (CTCAE v4.03) [44].

Many potential targets are currently being explored (Fig. 2). For instance, CAR-T cells targeting signaling lymphocytic activation molecule F7 (SLAMF7) and signaling lymphocytic activation molecule F3 (SLAMF3) for untreated and chemo-resistant MM patients have shown efficient killing in both in vitro and in vivo experiments. Compared with BCMA, SLAMF7 is a surface glycoprotein and is more evenly expressed on myeloma cells and less on B cells [152]. Patients who relapsed after receiving BCMA CAR-T cells may benefit from treatment with SLAMF7 CAR-T cells. SLAMF7 CAR-T cells demonstrated its anti-myeloma-killing effect in mouse models [153]. Clinical trials targeting SLAMF7 are ongoing (NCT03958656, NCT04499339). SLAMF3 CAR-T cells showed strong cytotoxicity in patients’ primary tumor cells and MM cell lines U-266 and RPMI-8226. In a xenograft mouse model, CAR-T cells also demonstrated strong anti-tumor effects and significantly prolonged the survival of mice [154].

.In 52% of MM patients, the LewisY antigen is present [155]. LewisY CAR-T cells have been shown to have the potential to persist and exert anti-tumor effects after infusion into patients (Fig. 2). Their specific efficacy is yet to be verified in further experiments [156].

According to several reports, G protein-coupled receptor class-C group-5 member-D (GPRC5D) could be an essential target (Fig. 2). Hair follicles seem to be the only normal tissue in which GPRC5D expression has been discovered outside of cancerous bone marrow plasma cells [157]. Researchers developed a humanized GPRC5D CAR-T cell and found that it could eradicate tumor cells in a mouse MM model of BCMA antigen escape without causing significant toxic side effects [157].

CD44v6 is considered one of the tumor stem cell markers. Preclinical studies have shown that CD44v6 CAR-T cells exhibit potent antitumor activity against MM but lead to a reduction in beneficial monocytes in mouse models [158] (Fig. 2).

Besides, New York esophageal squamous cell carcinoma-1 (NY-ESO-1) is an intracellular protein whose peptide could be presented on the cell surface by MHC molecules when it is ubiquitinated and degraded in the cell [159]. NY-ESO-1 is expressed in about 60% of MM patients, with higher levels in individuals with relapses, suggesting that NY-ESO-1 is intimately linked to MM disease progression [160]. In the context of HLA-A*02:01, CARs that recognize the NY-ESO-1 immunodominant peptide 157–165 were made to redirect autologous CD8(+) T cells to NY-ESO-1(+) MM cells. Preclinical trials confirmed the targeting effect, cytokine secretion, and ability to induce immune memory in NY-ESO-1 CAR-T cells [161].

Given that NKG2DL is expressed in MM malignant cells but not in healthy tissues, it has huge prospects for clinical application (Fig. 2). However, existing NKG2DL CAR-T cells have limited amplification and persistence in MM patients. For improved clinical efficacy, more research is needed to improve NKG2DL CAR-T cell expansion [162].

Interestingly, it has been shown that CD126 CAR-T cells infiltrate, expand, and kill tumor cells in a MM xenograft model without producing toxic effects, suggesting its great potential [163].

Researchers have created nanobodies against CD38 and constructed CD38 CAR-T cells from them (Fig. 2). The cells showed strong toxic effects against CD38+ MM cell lines (LP-1, RPMI 8226, OPM2, MOLP8, and primary MM cells from patients) and inhibited tumor growth in mice inoculated with RPMI 8226 cells [164]. However, it should be borne in mind that CD38 is also expressed at moderate levels in hematopoietic progenitor cell subpopulations and some normal hematopoietic cells [165].

CD138 is a primary diagnostic marker for MM and is a desirable target for the treatment of MM [166] (Fig. 2). Nonetheless, CD138 CAR-T cells may also attack normal skin and mucosal tissues [167].

CD56 is a possible immunotherapeutic target strongly expressed by malignant plasma cells in 70% of MM patients [168] (Fig. 2). The expression of CD56 on the central and peripheral nervous system has raised neurotoxicity concerns.

Given that MM is phenotypically heterogeneous, a single CAR-T treatment targeting only one antigen is challenging to attain long-term CR. Bispecific CAR-T cells targeting BCMA and CD38 were found to lead to clinical responses and minimal residual disease negative in 87% of MM patients in a phase 1 experiment (ChiCTR1800018143, 23 participants). Grade 3–4 hematological toxicity is more common in patients, rarely reaching grade 3 CRS, without neurological symptoms (CRS, Lee 2014 criteria; other adverse events, CTCAE v5.0) [169]. In another phase 2 trial (ChiCTR1800017051, 22 participants), the treatment resulted in CR in 55% of patients, and 27.3% of patients experienced an adverse event more significant than or equal to grade 3 (CRS, American Society for Transplantation and Cellular Therapy criteria; other adverse events, CTCAE v4.03) [170]. CD19/BCMA CAR-T cell therapy showed promising results in a phase 2 trial (ChiCTR-OIC-17011272, 62 participants), with CR or better outcomes observed in 60% of patients, CRS in 95% of patients, of which 10% were grade 3 or higher, and neurotoxic events in 11% of patients, of which 3% were grade 3 or higher (CRS, Lee 2014 criteria; other adverse events, CTCAE v4.03) [171]. Research on multi-specific CAR-T cells may lead to a breakthrough in the treatment of MM.

CAR-T cell therapy offers great promise in treating patients with hematological malignancies. Although there is still much room for development, it is currently showing exciting trends in B-ALL, MM, and B-NHL, especially in B-ALL and B-NHL. CD19 CAR-T cells have achieved excellent results in many cases of blood cancer. However, CAR-T cell therapy needs further exploration to treat patients with T-cell malignancies. The drug resistance of cancerous tissues to CAR-T cells and the possible side effects of treatment, such as severe inflammatory toxicity, are issues that need further research. Multi-target-specific CAR-T cells are currently the most commonly used treatment against drug resistance. HSCT and immunoglobulin transplantation could partially reduce the side effects of treatment, but they may have other adverse effects and may not be ideal solutions.

The most frequent CAR-T cell therapy targets in hematological malignancies are listed (Fig. 2).

## Solid tumor

### Breast cancer

Current evidence suggests that breast cancer (BC) accounted for 11.7% of all cancer types in 2020, surpassing lung cancer as the most prevalent cancer [172]. Recent advances in therapeutic approaches have improved BC patient survival and quality of life, but mortality rates remain high due to drug resistance limiting efficacy. Some members of the receptor tyrosine kinase (RTK) family and cell surface proteins are the primary targets for CAR-T cells to treat BC. Other targets include immune checkpoint, Ephrin type-A receptor (EphA) 10, stress ligands, disialoganglioside, and serum tumor markers [173].

**Fig. 3:**
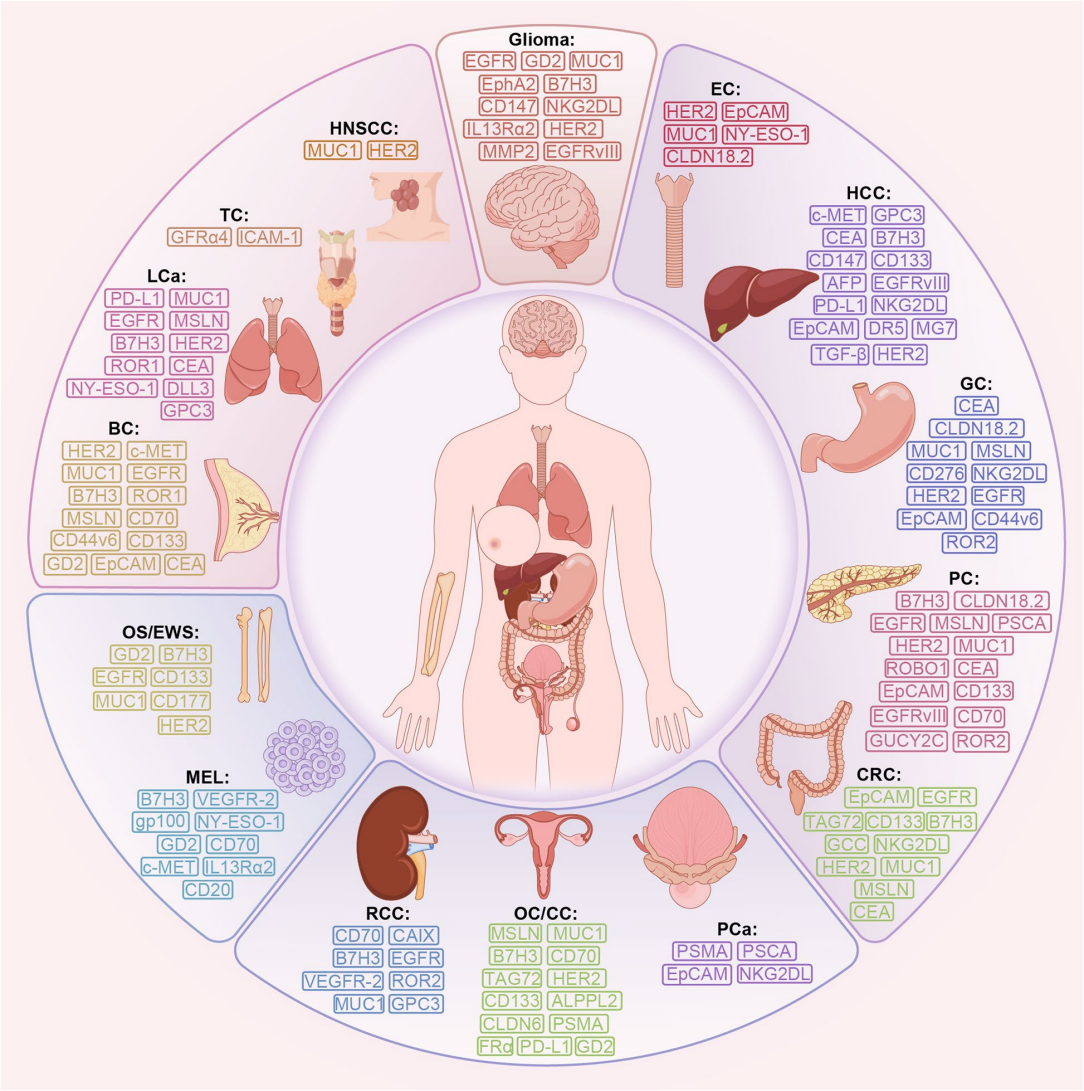
Common CAR-T cell therapy targets in solid tumors. EGFR: epidermal growth factor receptor; GD2: ganglioside2; MUC1: mucin 1; NKG2DL: natural killer group 2 member D ligand; HER2: human epidermal growth factor receptor 2; HNSCC: head and neck squamous cell carcinoma; TC: thyroid cancer; ICAM-1: intercellular adhesion molecule-1; LCa: lung cancer; PD-L1: programmed death-ligand 1; MSLN: mesothelin; ROR: receptor tyrosine kinase like orphan receptor; CEA: carcinoembryonic antigen; NY-ESO-1: New York esophageal squamous cell carcinoma-1; DLL3: delta-like ligand 3; BC: breast cancer; OS: osteosarcoma; EWS: Ewing’s sarcoma; MEL: melanoma; VEGFR-2: vascular endothelial growth factor receptor 2; RCC: renal cell carcinoma; CAIX: carbonic anhydrase IX; GPC3: glypican-3; OC: ovarian cancer; CC: cervical cancer; ALPPL2: alkaline phosphatase placental-like 2; PCa: prostate cancer; PSMA: prostate-specific membrane antigen; PSCA: prostate stem cell antigen; CRC: colorectal cancer; PC: pancreatic cancer; CLDN: claudin; GC: gastric cancer; GUCY2C: Guanylyl cyclase C; HCC: hepatocellular carcinoma; EC: esophageal cancer

Five RTKs, including human epidermal growth factor receptor 2 (HER2), epidermal growth factor receptor (EGFR), c-mesenchymal-epithelial transition factor (c-MET), ROR1, and AXL, are currently known to be targeted by CAR-T cells in BC to elicit therapeutic potential (Fig. 3). CAR-T cells have functioned in various preclinical BC models using these antigens (HER2, EGFR, c-MET, ROR1, AXL) as targets [174–178]. Four RTK targets have started clinical studies, including HER2, EGFR, c-MET, and ROR1. Currently, mRNA electroporation is considered the safest gene transduction method in T cells. The mRNA encoding the target gene is introduced into the cytoplasm by electroporation. It is also modified to increase stability and long-term expression. Although mRNA technology is efficient and easy to design in terms of transducing CARs compared to other transduction techniques, it also has a short lifespan [179]. In a phase 1 clinical trial, 3 × 10^7^ or 3 × 10^8^ c-MET CAR-T cells constructed via mRNA were administered to six patients with metastatic BC and showed well-tolerated results with inflammatory responses. None of the patients experienced more than grade 1 study drug-related adverse reactions (CTCAE v4.0). Only three patients developed grade 1 erythema (CTCAE v4.0). This trial used an intratumoral injection route, c-MET CAR-T cells, and an anti-tumor response could be detected at the injection site (NCT01837602) [176]. The median follow-up was ten months (range 3–28 months), with two patients progressing, three patients dying from the disease, and one with stable disease. The latest clinical trials of CAR-T cell therapies targeting HER2 (NCT05007379), EGFR (NCT05341492), and ROR1 (NCT05274451) for BC have begun between 2021 and 2022. But all clinical trials of CAR-T cells targeting HER2, EGFR, and ROR1 for BC have not reported results.

Overexpressed proteins on the surface of BC cells suggest that they may be good candidates for CAR-targeted therapeutic interventions. At present, they mainly include mucin 1 (MUC1), mesothelin (MSLN), CD70, CD133, CD44v6, epithelial cell adhesion molecule (EpCAM), chondroitin sulfate proteoglycan 4 (CSPG4), intercellular adhesion molecule-1 (ICAM-1), Tumor endothelial marker 8 (TEM8), a trophoblast cell surface antigen 2 (TROP2), and folate receptor alpha (FRα) [173, 176, 180–189] (Fig. 3). Only six surface proteins have entered clinical studies, including MUC1 (NCT02580747), MSLN (NCT02414269), CD70 (NCT02830724), CD133 (NCT02541370), CD44v6 (NCT04430595), and EpCAM (NCT02915445). The above NCT numbers refer to each target’s most recently started or updated clinical trials. The safety and efficacy of CAR-T cell treatments targeting MSLN and EpCAM are evaluated in phase 1 clinical trials. The remaining CARs are in phase 1 and phase 2 clinical trials. In addition, the three CAR-T cells targeting MUC1 yielded heterogeneous effects in different clinical trials (NCT04020575, NCT04025216, and NCT02587689, respectively), targeting different structural domains of the cleaved form of MUC1, aberrant glycated MUC1, and entire MUC1 [173]. No preclinical evidence for CD44v6 CAR-T cell therapy for BC could be found. Still, it has also entered clinical trials due to its proven anti-tumor capacity in other preclinical cancer models [190]. In contrast, several preclinical studies on the remaining ten cell surface protein targets (CD133, MUC1, MSLN, CD70, EpCAM, CSPG4, ICAM-1, TEM8, TROP2, FRα) have shown potential as CAR-T targets for the treatment of BC [180–189].

Target selection is one of the determinants of CAR-T cell efficacy. Therefore, researchers have been working hard to identify new targets. Recent preclinical studies reported CAR therapy targeting EphA10 as a promising strategy for treating triple-negative BC [191].

Li et al. designed a novel PD-L1-targeted shark VNAR single-domain CAR-T cell. Shark VNAR is small that could bind epitopes difficult to conventional antibodies. They found that this type of CAR-T cell could lyse cancer cells in breast and liver cancer models by targeting immunosuppressive microenvironment antigen (PD-L1) [192].

Preclinical trials of ganglioside 2 (GD2), protein tyrosine kinase 7 (PTK7), and NKG2DL as CAR-T cell therapy targets also showed anti-BC activity [177, 193–195] (Fig. 3). Clinical trials on the safety and efficacy of GD2 CAR-T cells are underway (NCT04430595, NCT03635632).

No preclinical study of CAR-T cells for BC has targeted the serum tumor marker carcinoembryonic antigen (CEA), while a clinical trial of CAR-T cell therapy targeting CEA to remedy BC subjects is underway (NCT04348643) (Fig. 3).

In conclusion, although many institutions have registered many BC clinical trials of CAR-T cells over recent years, few results have been published. Accordingly, more researches are indispensable to validate and compare the effectiveness of different targets.

### Lung cancer (LCa)

LCa is one of the most prevalent tumors globally, with a high degree of malignancy and poor prognosis [172]. Based on histological features, LCa could be divided into non-small cell lung cancer (NSCLC), which accounts for 85% of diagnosed LCa cases, and small cell lung cancer (SCLC) [196]. Although the prognosis of LCa has improved significantly in recent years with targeted and immune drugs, it is still unsatisfactory, and the mortality rate is high [196]. Mounting evidence suggests CAR-T cell therapy is effective in treating NSCLC [197].

According to the literature, CAR-T cells’ most common targeted antigens in NSCLC are EGFR, MSLN, CEA, PD-L1, ROR1, B7H3, MUC1, HER2, and Delta-like ligand 3 (DLL3) [177, 198–204] (Fig. 3). These targets have been proven to have tumor-suppressive effects in preclinical models and applied in clinical trials. Clinical studies of CAR-T therapy for LCa have published outcomes from a phase 1 trial in which EGFR CAR-T cells generated by the piggyBac transposon system were well tolerated by all patients with advanced relapsed/refractory EGFR(+) NSCLC (*n* = 9), with no reports of grade 4 adverse events or severe CRS (NCT03182816, CTCAE v5.0) [205]. The piggyBac transposon system was chosen to construct CAR-T cells in NCT03182816 because it is more straightforward and cost-effective than viruses. One patient had a sustained response of more than 13 months, while six and two patients had stable disease and progressive disease, respectively. The median progression-free survival (mPFS) was 7.13 months, with an mOS of 15.63 months. The above results indicate that this therapeutic approach is safe and effective [205]. Other than this, no remaining clinical trial results were reported. MSLN has the eleven LCa clinical trials registered on Clinicaltrials.gov, but no experimental results are currently available. CAR-T cells targeting MSLN killed NSCLC cells and exhibited greater anti-tumoural capacity than unmodified T cells in mouse models. Still, persistence is an issue that needs to be addressed [206].

DLL3 is considered a novel target for SCLC treatment; increased expression of DLL3 was found in SCLC and other neuroendocrine tumors, with lower expression levels in most normal tissues [207] (Fig. 3). However, clinical trials of DLL3 CAR-T cells for treating relapsed/refractory small cell LCa have been suspended due to the absence of active subjects in the trial (NCT03392064).

The NCT numbers of the latest initiated or updated clinical trials for the remaining targets are listed here for reference: CEA (NCT04348643), PD-L1 (NCT03330834), ROR1 (NCT05274451), EGFR/B7H3 (NCT05341492), MUC1 (NCT05239143), and HER2 (NCT04660929).

MAGE-A1 antigen, glypican-3 (GPC3), FRβ, CD44v6, CD133, c-MET, Olfactory receptor 2H1 (OR2H1), CD47, GD2, CD147, prostate stem cell antigen (PSCA), Fibroblast activating protein (FAP), EphA2 and PTK7 are also expected to be targets of CAR in the context of LCa, with preclinical studies completed [190, 195, 203, 204, 208–218] (Fig. 3). However, the relevant clinical trials are still not registered to be conducted.

Likewise, CLEC14A is an overexpressed tumor endothelial marker with relatively negligible physiological expression in normal endothelial cells. CLEC14A-redirected CAR-Ts sufficiently released IFN-γ and enforced anti-tumor effects in vitro. The intelligence behind the targeting of CLEC14A is that it is a glycoprotein with elevated expression in various solid tumors [219]. The treatment of CLEC14A-redirected CAR-Ts significantly inhibited tumor growth in Lewis lung carcinoma, Rip-Tag2, and mPDAC mouse models without signs of toxicity [220]. No registered clinical trials are using CLEC14A CAR-T cells to treat LCa.

Although CAR-T cell immunotherapy has demonstrated potential in various preclinical models of LCa, the pool of targeting antigens still needs to be expanded. More novel approaches need to be applied to find them. For example, antigens with a significantly higher expression on the surface of tumor cells could be used as targets. CXCR4 is highly expressed in LCa and is expected to be a novel target for NSCLC [221].

Anti-NY-ESO-1 TCR-transduced T cells have been shown to kill LCa cells (A549-A2-ESO) and depress the growth of tumors in xenograft mice models, but CAR-T cell clinical trials targeting this antigen have not been conducted [222].

### Prostate cancer (PCa)

PCa is the most common tumor of the male genitourinary system, with more than 1.4 million cases and over 375,000 deaths worldwide [172]. Fatal metastatic debulking-resistant PCa is a late-stage sequela with only a median survival of 10 months to 21.7 months, a 30% five-year survival rate, and a poor prognosis. Although radiation, chemotherapy, and hormonal therapies have significantly progressed in treating PCa, limited treatment is available for patients with advanced diseases [223].

It is widely thought that prostate-specific membrane antigen (PSMA) is an attractive target that could be used to treat PCa (Fig. 3). PSMA is predominantly expressed in the healthy prostate and, to a lesser extent, in other tissues, including the intestine, brain, kidney, lacrimal gland, and salivary gland [224]. Notably, PSMA is expressed in almost all primary and metastatic PCas [225]. However, PSMA-directed CAR-T cells are less effective in lysis therapy. Indeed, CAR-T cells must overcome the immune-cold TME and efficiently transport and penetrate the site of tumor metastasis [226, 227]. Christopher C Kloss et al. improved the efficacy and safety by blocking transforming growth factor-β (TGFβ) signaling in T cells, allowing CAR-T cells to work better in PCa models [228]. They conducted a concurrent clinical trial with four therapeutic dose levels of TGFβ-insensitive armored CAR-T cells administered to 13 subjects (NCT03089203). Five patients were observed to develop grade ≥ 2 CRS, including one with prostate-specific antigen (PSA) reduction > 98%, and one died after experiencing grade 4 CRS complicated by sepsis (American Society for Transplantation and Cellular Therapy criteria). After adoptive cellular transfer, three other patients were found to have ≥30% reduction in PSA and CAR-T cell failure with simultaneous upregulation of multiple TME local suppressor molecules. The median of 15.9 months was good [228]. In conclusion, the clinical use of combining TGFβ blockade and PSMA CAR-T cells is promising and generally secure, and therapeutic approaches in combination with targeting inhibitory factors should be feasible. In addition, Claudia Arndt et al. built a modular platform called UniCAR. Here, they constructed a UniCAR epitope in combination with PSMA-11 to generate a compound that redirects UniCAR T cells to tumor cells. The advantage of UniCAR T cells is that bispecific bridging molecules, called target modules, could mediate them and do not interact directly with tumor cells like conventional CAR-T cells [225]. This finding provides a good tool and direction for developing diagnostic imaging and targeted therapy for PCa.

PSCA has gained significant attention as an important marker for bladder, prostate, and pancreatic cancers [227] (Fig. 3). Currently designed PSCA CAR-T cells have shown substantial antitumor effects in disease models of prostate and pancreatic cancers [229, 230].

Deng et al. demonstrated that in EpCAM CAR-T (Fig. 3), human peripheral blood lymphocytes have antitumor activity against PCa [231].

IL-7 was found to have an enhanced effect on NKG2DL CAR-T cell immunotherapy, which provides a therapeutic approach [232] (Fig. 3).

The clinical trials targeting PSCA (NCT03873805, NCT02744287), EpCAM (NCT03013712), and NKG2DL (NCT04107142) have not reported their results.

In addition, CAR-T cells targeting CEA, B7H3, MUC1, and CD126, respectively, have been found to play different roles, although all have antitumor activity [163, 233–235]. In addition to the therapies mentioned above, CAR-T cells with an inducible “ON” safety switch have recently been designed and shown to improve outcomes [236]. NCT04249947 is an ongoing phase 1 study targeting PSMA CAR-T cells using rimiducid as the “ON” or safety switch activator, which controls initiation and activation and could reduce toxic responses in a controlled manner.

### Colorectal cancer (CRC)

CRC is the second leading cause of cancer-related death [172]. Disease control or cure could be achieved through early detection by screening and good results with conventional therapies for localized tumors. However, metastatic CRC remains a tricky problem [237], and patients with metastatic CRC have been the focus of CAR-T cell therapy. The first trial of CAR-T cells for solid tumors was conducted in the 1990s. Patients with metastatic CRC were treated with CAR-T cells targeting TAG72 in two phase 1 trials, one by intravenous infusion and the other by hepatic artery infusion (Fig. 3). Difficulties in T-cell transport to metastatic sites were found, but their relative safety was also demonstrated [238].

Many antigens targeted by CAR-T cell therapies for CRC have been tested and validated in preclinical studies and clinical trials in recent years. CEA is the most promising target for disseminated CEA CRC (Fig. 3). Current evidence suggests that CEA is overexpressed as a serum marker in 98.8% of CRC tissues [239]. Therefore, CEA is considered an attractive target for CAR-T therapy in CRC. Several clinical trials have been conducted for CAR-T therapies targeting CEA. In a phase 1 clinical trial (NCT02349724), CAR-T cells targeting CEA were applied systemically in 10 patients with metastatic CRC. The treatment was effective and well-tolerated even at high dose levels. Seven patients who experienced the progressive disease in previous treatments were stabilized, two of whom were stable for more than 30 weeks, and two others experienced tumor shrinkage. No serious adverse events associated with CAR-T cell therapy have been observed [240]. The efficacy could also be enhanced by lymphodepletion with cyclophosphamide/fludarabine chemotherapy [240]. This trial suggested that the safety profile of CEA CAR-T cell therapy is good, with only mild and manageable adverse effects associated with CRS, which was demonstrated by another trial [241]. Even during long-term observation, the decrease in serum CEA levels was evident in most patients. However, CEA CAR-T cells have been reported to induce transient colitis because CEA is expressed on normal gut epithelial cells [242]. To address this issue, Mark et al. designed CEA Tmod cells using a CAR activated by CEA and an LIR-1-based inhibitory receptor triggered by HLA-A*02 [243]. These cells could harness the loss of HLA heterozygous genes in tumors to safely and effectively kill tumor cells. However, unlike CEA CAR-T cells, Tmod cells still could specifically target tumor cells in the presence of cells expressing HLA-A*02 [243]. However, there are no clinical trials of Tmod cells.

CD133 is highly expressed on many solid tumors, and CD133 is a marker for cancer stem cells (CSCs) and endothelial progenitor cells [244]. Clinical trials for CAR-T cells targeting CD133 have also been published (Fig. 3). An antitumor response was observed in phase 1 clinical trial, NCT02541370, that recruited 23 patients suffering from HCC (*n* = 14), PC (*n* = 7), and CRC (*n* = 2), treated with CD133 CAR-T cells [245]. Three achieved PR, 14 patients were SD, and the mPFS was 5 months. The 2 CRC patients had SD. More extended periods of disease stabilization could be observed after repeated infusions of cells and are more effective in patients who have achieved some efficacy after the first cell infusion [245]. The primary toxicity is hemoglobin/thrombocytopenia (≤ grade 3), which recovers spontaneously within 1 week (CTCAE v4.0).

The NCT numbers of the latest initiated or updated clinical trials for the remaining targets are listed here for reference: MUC1 (NCT05239143), MSLN (NCT05089266), EpCAM (NCT05028933), HER2 (NCT04660929), NKG2DL (NCT04550663) and GUCY2C (NCT05287165). But there are no reports on the results of these experiments.

The combination of regorafenib and EpCAM CAR-NK cells performs more effectively in human CRC models than monotherapy with CAR-NK cells or regorafenib [246].

CAR-T cell therapy targeting HER2 showed potent results in animal models of CRC [247] (Fig. 3). Still, it resulted in acute respiratory failure syndrome in a case report, highlighting the need for further improvements [248].

In addition, the antitumor efficacy of NKG2DL RNA CAR-T cells was confirmed in a mouse model of peritoneal metastasis of colon cancer [249] (Fig. 3).

Guanylyl cyclase C (GUCY2C) CAR-T cells designed by Magee et al. identified and killed CRC cells that endogenously express GUCY2C [250].

MSLN, MUC1, placental alkaline phosphatase (PLAP), c-MET, and Cadherin-17 (CDH17) are also promising targets in CAR-T cell therapies for CRC, validated in several preclinical trials [251–255] (Fig. 3).

### Gastric cancer (GC)

GC is a common malignancy globally, with gastric adenocarcinoma accounting for more than 90% of cases [256]. Despite the continuous improvement and innovation of therapeutic approaches for GC, treatment options for GC remain limited. CAR-T cell therapies are currently considered a promising therapeutic approach, with multiple target antigens that may be effective targets.

Claudin (CLDN) 18.2 was present in 70% of primary gastric adenocarcinomas and their metastases [257] (Fig. 3). In an ongoing, open-label, single-arm, phase 1 clinical trial, three different doses of CAR-T cells which aim at CLDN18.2 were employed for the treatment of CLDN18.2+ gastrointestinal cancers; 37 patients were treated, with 94.6% of patients experiencing grade 1 or grade 2 CRS but no serious adverse effects (NCT03874897, American Society for Transplantation and Cellular Therapy criteria); the ORR was 48.6%, and disease control rate (DCR) was 73.0%; the ORR and DCR of GC patients reached 57.1 and 75%, and the 6-month OS rate of GC patients reached 81.2% [258]. This finding corroborates the safeness and potency of CLDN18.2 CAR-T cells in CLDN18.2+ gastrointestinal cancers, especially in GC patients.

Many clinical trials of CAR-T cells targeting these targets (HER2, CEA, EpCAM, CLDN18.2, MSLN, MUC1, NKG2DL, EGFR, B7H3) have been registered and conducted [259] (Fig. 3). Clinical trials using CAR-T cells targeting ROR2 and CD44v6 for GC have also been reported to validate their feasibility and safety, but preclinical studies are scarce (NCT03960060, NCT04427449). However, Other than the CLDN18.2 results mentioned above, no other GC clinical trial results have been published.

Significantly, EpCAM is overexpressed in more than 90% of GC and has aroused interest due to its homogeneous expression [260]. In preclinical studies, CAR-T cell therapies targeting EpCAM have demonstrated antitumor effects [261].

HER2 is overexpressed in 10–20% of GCs and could affect CSCs (Fig. 3). Preclinical studies demonstrated that CAR-T cells targeting HER2 could recognize and lyse GC cells (N87, 7901, AGS, HGC27, MGC803, BGC823, MKN45, primary GC cells) with high affinity and significantly inhibited the in vivo tumorigenic capacity of CSCs [262].

CEA is also a potential target of CAR-T cells for treating GC since the high expression on the tumor cells and combining CEA CAR-T cells with recombinant human IL-12 significantly inhibited tumor growth [263] (Fig. 3).

In addition, the potential of CAR-T cells targeting MUC1, MSLN, NKG2DL, EGFR, and B7H3 has been validated in preclinical studies [264–268].

The NCT numbers of the latest initiated or updated clinical trials for these targets are listed here for reference: HER2 (NCT04660929), CEA (NCT05396300), EpCAM (NCT05028933), MUC1 (NCT05239143), MSLN (NCT03941626), EGFR (NCT03740256), B7H3 (NCT04864821), and NKG2DL (NCT04550663).

Indeed, CAR-T cell therapy still faces many problems, and finding new targets is the key to improving the therapeutic efficacy. Researchers substantiated the effectiveness of CAR-T cells targeting PSCA, FRα, PD-L1, c-MET, CD133, CDH17, ICAM-1, and urokinase plasminogen activator surface receptor (uPAR) in GC models in the last 2 years [255, 269–275].

In addition, antigens such as B7H6, ARP2/3, NRP-1, DSC2, AE1/2, TAG72, and CA19–9 have been suggested as possible targets for GC treatment with CAR-T cells [259, 276] (Fig. 3).

To further improve the efficacy, Zhao et al. designed bispecific Trop2/PD-L1 CAR-T cells with a significantly enhanced ability to inhibit tumor growth by intratumoral injection [277]. Because Trop2 and PD-L1 are highly expressed in various solid tumors, the bispecific cells could target two antigens (Trop2/PD-L1) with high specificity and be capable of blocking the PD-1/PD-L1 signaling pathway.

### Liver cancer

Liver cancer currently ranks sixth in incidence among common malignancies worldwide and is the third leading cause of cancer-related deaths [172]. 85–90% of primary liver cancers are hepatocellular carcinoma (HCC), and surgery is often not indicated since most patients are diagnosed with HCC at an advanced stage [278]. Nowadays, targeted therapy and immunotherapy have achieved good results compared to the previous ones, but the prognosis of liver cancer is still poor.

Glypican-3 (GPC3) enhances HCC cell proliferation through the Wnt/β-catenin pathway and is the most commonly used target site of CAR-T cell therapies for HCC (Fig. 3). GPC3 has been documented in 72% of HCC patients, and 53% had significantly high serum GPC3 levels [279]. The high specificity and sensitivity of GPC3 have made it a target for diagnosing and treating HCC. Jiang et al. showed that CAR-T cells targeting GPC3 could inhibit tumor growth significantly in an in vivo model [280]. Many clinical studies of CAR-T cells targeting GPC3 for liver cancer are underway. In published phase 1 trial results, GPC3 CAR-T cells that could secrete IL-7 and CCL19 were injected intratumorally in a patient with advanced HCC. The tumor was eliminated within 30 days (NCT03198546). The patient developed severe fever, and no other serious side effects were observed [281].

CEA is also a target that has been studied in-depth (Fig. 3). In a phase 1b HITM-SIR clinical trial, Steven C. Katz et al. used CEA CAR-T cells to treat six patients with CEA+ liver metastases. CEA CAR-T cells infused via the hepatic artery were well tolerated. No grade 4 or 5 toxicities, severe CRS, or neurotoxicity were observed (CTCAE v4.03). And biological responses were demonstrated following conventional therapy (NCT02416466) failure with mOS for 8 months [282]. This clinical trial illustrates that CEA CAR-T cells infused by this delivery method could effectively treat liver cancer.

In addition to improving the CAR-T architecture, targeting CSCs is a strategy since CSCs play an essential role in promoting tumors. CD133 is considered a marker of CSCs (Fig. 3). In a single-arm, open-label phase 2 clinical trial, 21 advanced HCC patients were infused with CD133 CAR-T cells (NCT02541370). One was in PR, 14 individuals had SD for 2 to 16.3 months, and 6 had PD [283]. Four patients developed grade 3 hyperbilirubinemia, two had grade 3 anemia, and no other serious adverse events occurred (CTCAE v4.0). These findings indicate that CD133 CAR-T cells have antitumor efficacy and low toxicity in patients with advanced HCC [283]. NCT02541370 is a phase 1/2 clinical trial with phase 1 and phase 2 results published separately. The early results mentioned in the CRC section of the text are from the phase 1 trial, while the subsequent phase 2 trial report only mentions long-term clinical outcomes in HCC patients [245, 283].

In addition, CAR-T cells against DR5 (NCT03941626), MG7 (NCT02862704), HER2 (NCT04842812), and TGFβ (NCT03198546) are also being evaluated in clinical trials for the treatment of liver cancer, and the results are expected to be announced soon.

As molecular technology advances, more antigens are considered potential targets for CAR-T cells to treat liver cancer (Fig. 3). For example, CAR-T cells targeting AFP, EGFRvIII, B7H3, EpCAM, MUC1, NKG2DL, PD-L1, and CD147 were demonstrated in preclinical studies [189, 284–289]. Many clinical trials for these targets have also been registered and conducted. But there are still no reports about results. We list here the NCT numbers of the most recently initiated or updated clinical trials for these targets: AFP (NCT03253289), EGFRvIII (NCT03941626), B7H3 (NCT05323201), EpCAM (NCT03013712), MUC1 (NCT04842812), NKG2DL (NCT04550663), PD-L1 (NCT03672305), and CD147 (NCT03993743).

CD44 is a transmembrane glycoprotein that critically mediates cell adhesion, interaction, and migration [290]. scFv-based CD44-redirected CAR-Ts were potentially cytotoxic towards the HCC cell lines (Hep3B2, MHCC97H, SMMC-7721, HepG2, PLC8024) and secreted elevated levels of IL-2, IFN-γ, and TNF-α. Moreover, CD44-redirected CAR-Ts showed no signs of toxicities toward healthy tissues and significantly inhibited tumor growth in CD44-positive HCC xenograft mice [291]. There are no relevant clinical trials available.

To improve the therapeutic effect of CAR for HCC, researchers have developed novel strategies: combining immune checkpoint PD-L1 with CAR-T therapy and designing CAR-T cells targeting c-MET and PD-L1 simultaneously (Fig. 3). These dual-targeted T cells showed more vigorous growth inhibitory activity than single-targeted cells but also enhanced the ability of activated T cells to proliferate and produce INF-γ [292]. Another novel inducible CAR-T cell could control CAR expression. For example, the third-generation gene expression system, Tet-On 3G, could reversibly turn gene expression on or off, achieved by doxycycline (Dox) [289]. Experiments have proved that (Dox+) Tet-CD147 CAR-T cells generated by Tet-On 3G exhibited more potent cytotoxic effects and cytokine secretion than (Dox-) Tet-CD147 CAR-T cells [289].

### Esophageal cancer (EC)

EC, which could be classified as esophageal squamous cell carcinoma (ESCC) and esophageal adenocarcinoma (EAC), is cancer with limited treatment options and a poor prognosis. EC ranks seventh in terms of incidence (604,000 new cases) and sixth in mortality overall (544,000 deaths) [172]. CAR-T cell therapy represents a potential therapeutic approach for EC. However, there are still no reports of clinical trial results on EC treatment with CAR-T cells.

B7H3 is strongly and uniformly expressed in ESCC and EAC malignant cells but rarely in healthy tissue. B7H3 CAR-T cells effectively kill ESCC tumor cells in human ESCC cell lines (EC109, KYSE150, TE-1, KYSE450, KYSE510, TE-7) and xenograft mouse models. CAR-T cells that induce tumor regression in a xenograft model prolong the survival of mice [293]. Tandem CAR-T cells targeting CD70 and B7H3 also exhibited anti-EC function [189].

HER2 was highly expressed in ESCC but at limited levels in normal esophageal tissues (Fig. 3). HER2 CAR-T cells demonstrated good therapeutic efficacy in HER2+ ESCC tumor cell lines (ECA109, TE-1) and xenograft mouse models [294].

In addition, EpCAM overexpression is associated with low survival in patients with ESCC [295] (Fig. 3). Research on CAR-T cells targeting HER2 and EpCAM-positive cancer is underway (NCT03740256, NCT03013712).

NY-ESO-1 TCR-engineered T cells have also been used in a clinical trial of EC (NCT03941626) (Fig. 3). However, no detailed information about this trial has been reported yet.

To enhance the activation and proliferation of CAR-T cells in solid tumors, Zhang et al. designed JAK-STAT domain-enhanced MUC1 CAR-T cells, which were found to induce the elimination of EC [296] (Fig. 3). This kind of CAR-T cell activated cytokine signaling pathways simultaneously while targeting MUC1. This is because the CAR structure of these cells integrated the IL2 receptor and the YXXQ motif of binding to STAT3, providing cytokine signals. A clinical trial using MUC1 CAR-T for EC is underway (NCT03706326).

### Pancreatic cancer (PC)

PC is a malignant digestive system tumor with a distinct immunosuppressive environment. Due to the poor prognosis, the number of deaths from PC (466,000) is almost the same as the number of cases (496,000), which is the seventh most significant cause of cancer death in men and women [172].

The in-depth studied CAR targets in PC are MSLN, EGFR, HER2, CEA, and CD133, and the results of published clinical trials for these targets are summarized below (Fig. 3). A phase 1 study evaluated the toxicity and activity of CAR-T cells against MSLN in patients with malignant pleural mesothelioma (*n* = 5), ovarian cancer (n = 5), and pancreatic ductal adenocarcinoma (n = 5) (NCT02159716) [297]. In another clinical trial (NCT01897415), two of the six patients with chemotherapy-refractory metastatic pancreatic ductal adenocarcinoma were stable after treatment, and no patient developed dose-limiting toxicity, CRS, or neurological symptoms. The trial found that RNA CAR-T cells did not persist and lacked targeting toxicity [298]. Therefore, this clinical trial (NCT02159716) used MSLN CAR-T cells transduced with a lentiviral vector to increase CAR-T cell levels in peripheral blood. The results showed that MSLN CAR-T cells transduced with a lentiviral vector could expand in the blood, and pretreatment with cyclophosphamide promoted cell expansion. All patients well tolerated the therapy, but no additional clinical reactions were observed except for stable disease (11/15). A total of 20 grade 3 or higher adverse events were observed (CTCAE v4.0) [297]. This finding may be because only 3 of the 15 patients expressed MSLN on > 75% of the tumor cells, suggesting that a certain percentage of tumor cells expressing the target may be required to achieve significant clinical activity. This finding is consistent with observations from NCT01897415, where the lack of MSLN expression on tumor cells surface was associated with the limited efficacy of MSLN CAR-T cell therapy [298]. Therefore, patients could be screened prospectively for surface antigen expression to improve the efficiency of subsequent clinical trials. In another trial, Pang et al. designed a CAR-T cell (MSLN-7 × 19 CAR-T) targeting MSLN capable of secreting IL-7 and CCL19 in a patient with advanced PC. The tumor almost completely disappeared 240 days after intravenous infusion of MSLN-7 × 19 CAR-T cells (NCT03198546). No grade 2–4 adverse events or significant complications were observed [281].

EGFR is also a well-studied target in PC, and Yang et al. conducted a phase 1 clinical trial administering EGFR CAR-T cells to patients with metastatic PC and showed that the cells were safe and effective (NCT01869166) (Fig. 3). Of the 14 patients, 4 experienced a partial response for 2–4 months, and eight were stable for 2–4 months. Reduced EGFR expression on tumor cells was observed in patients with stable disease. Grade ≥ 3 adverse events included fever, nausea, etc., and were reversible (CTCAE v4.0) [299].

In addition, a phase 1 clinical trial targeting HER2 demonstrated the clinical activity of HER2 CAR-T cells in eleven enrolled patients with advanced biliary tract cancers and PC (NCT01935843) (Fig. 3). The mPFS was 4.8 months (range, 1.5–8.3 months); most adverse events were mild or moderate [300].

A patient with liver metastases secondary to stage IV pancreatic adenocarcinoma received locally infused CEA CAR-T cells (NCT02850536) (Fig. 3). The biological activity was demonstrated at 23.2 months, comparing favorably to the median survival time of 5 months for most stage IV patients. No serious adverse events above grade 3 were observed, indicating the treatment is safe [301].

In a phase 1 trial mentioned earlier (NCT02541370), two of the nine patients (7 of PC, 2 of CRC) treated with CD133 CAR-T cells achieved an OR, two achieved a PR, and five were stable, all with grade 2–4 lymphopenia (CRS, Lee 2014 criteria; other adverse events, CTCAE v4.0) [245].

These results provide data and experience into the future development of CAR-T strategies for treating PC.

Moreover, several other potential targets have been studied (Fig. 3), including B7H3, PSCA, MUC1, roundabout homolog 1 (ROBO1), EpCAM, CLDN18.2, CD318, TSPAN 8, and FAP. Some studies have shown that CAR therapies against these targets could inhibit tumor growth in mouse models of PC [302–308]. Only B7H3, PSCA, MUC1, ROBO1, EpCAM, and CLDN18.2 have entered PC clinical studies, but there are still no reports. We list here the NCT numbers of the most recently initiated or updated clinical trials for these targets: B7H3 (NCT05143151), PSCA (NCT03267173), MUC1 (NCT05239143), ROBO1 (NCT03941457), EpCAM (NCT05028933), and CLDN18.2 (NCT04404595). Especially, NCT03941457 evaluates the safety and efficacy of ROBO1 CAR-NK cell immunotherapy for PC. In particular, enforced expression of CXC chemokine receptor type 6 (whose ligands are highly expressed in human and murine PC cells and tumor-infiltrating immune cells) in T cells could enhance the recognition and lysis of PC cells because we know chemokines and their receptors are essential for the migration and homing of lymphocytes [309].

EGFRvIII, GUCY2C, ROR2, and CD70 are promising targets (Fig. 3), and related clinical trials have been conducted (NCT03267173, NCT05287165, NCT03960060, NCT02830724). Indeed, further studies on these targets could provide a foothold for optimizing CAR-T cell therapies.

Podocalyxin (PODXL), also called TRA-1-60, is a type I membrane-bound glycoprotein. A murine PODXL-specific CasMab was successfully developed with exclusive reaction with the PODXL-overexpressing GBM cell line (LN229) and PC cell line (MIA PaCa-2). Then, a core fucose-deficient mAb, 60-mG2a-f, was developed by conferring augmented antibody-dependent cellular cytotoxicity (ADCC) to CasMab. 60-mG2a-f exhibited remarkable anti-tumor capacity in the MIA PaCa-2 xenograft mouse model of PC, suggesting a promising targeted immunotherapy approach [310]. There are no relevant clinical trials available.

### Melanoma (MEL)

MEL is the most malignant skin cancer, formed mainly by the malignant proliferation of melanogenic cells (called melanocytes) in the skin. Several studies of different CAR-T cells have provided a theoretical basis for related clinical studies of MEL, including CD16, CD126, CD70, B7H3, HER2, Vascular endothelial growth factor receptor 2 (VEGFR-2), gp100/HLA-A2 complex, NY-ESO-1, CD20, mitochondrial-associated cysteine-rich protein, PD-L1, CSPG4, GD2 and GD3 have been investigated as promising targets of CAR-T cells [311–320] (Fig. 3).

For example, CD126 CAR-T cells showed potent tumor-suppressive activity in a metastatic MEL xenograft mouse model.

CSPG4 is overexpressed in most MEL cancer cell lines. One study designed a CSPG4 CAR-NK cell that released fewer cytokines than CAR-T cells but was capable of killing MEL cells and improving cytotoxicity [321].

αvβ3 integrins are reportedly overexpressed in many cancers, including MEL, breast, prostate, and PCs, and it plays a role in cancer survival and metastasis. Results of preclinical studies suggest that targeting αvβ3 integrins hold promise for treating MEL patients [322].

Many clinical trials have been registered and conducted for specific targets of MEL treatment with CAR-T cells, including VEGFR-2, GD2, c-MET, CD70, gp100, CD20, interleukin-13 receptor subunit alpha-2 (IL13Rα2), B7H3, and bispecific B7H3/CD19 (Fig. 3). Although clinical trials of CAR-T cells targeting IL13Rα2, c-MET, and CD20 for MEL are in progress to verify its feasibility and safety, few preclinical studies have been conducted. Most clinical trials have not been completed, or results have not been published. The following are all the published clinical trial results of CAR-T cell therapy for MEL. In phase 1 of the CARPETS trial (ACTRN12613000198729), GD2-positive metastatic MEL patients receiving CAR-T cell therapy showed upregulated LAG-3 and PD-1 expression in stimulated CAR-T cells [316]. Therefore, combined immune checkpoint blockade therapy may enhance the effect of CAR-T cell therapy. In a phase 1/2 study of 24 metastatic MEL patients (NCT01218867), no therapeutic effect was observed in patients treated with a combination of IL-2 and CAR-T cells targeting VEGFR-2, and 23 patients experienced adverse events, 5 of which were severe. In summary, the published clinical study results are not ideal.

CAR-T cell therapy could inhibit tumor growth through anti-angiogenic effects and direct cell killing while targeting multiple antigens simultaneously could also enhance efficacy. And these strategies have been applied to studies related to MEL. A study reported the simultaneous infusion of T cells specific for tumor antigen (gp100, TRP1, or TRP2) and CAR-T cells against the mesenchymal vascular system (VEGFR-2), which yielded a synergistic effect on a mouse model of MEL eradication and improved tumor-free survival versus treatment with either cell type alone or with T cells that co-express the two target molecules [314] (Fig. 3). To counter the immune escape mechanism of tumor cells, a specific combination of TCR against gp100 and CAR against CSPG4 was used [323]. Then CD8+ T cells expressing these two additional receptors were generated using electroporation receptor-encoding mRNA technology, and a strong effect was shown [323]. In addition, a Tandem CAR-T cell targeting both CD70/B7H3 yielded a significant cytolytic result in MEL model [189].

Overall, more studies are needed to assess the safety and efficacy of CAR-T cell therapeutic targets for MEL.

### Ovarian cancer (OC)

The prognosis for OC is the poorest, and its fatality rate is the greatest of all gynecologic cancers. It is among the most prevalent forms of cancer found in women around the entire globe [324].

MSLN is the most intensively studied target and has the most significant number of relevant clinical trials. Studies have identified that MSLN is overexpressed in more than 75% of high-grade serous ovarian carcinoma tumors [325]. Preclinical studies focused on using MSLN CAR-T cells in subcutaneous or in situ mouse OC transplantation models and found them to inhibit tumor growth [326]. In a phase 1/2 clinical study (NCT03615313), a relapsed patient with epithelial OC was treated with αPD-1-meso CAR-T cells which contain MSLN CAR and antibody for PD-1 in combination with apatinib (a drug that inhibits angiogenesis), the patient experienced PR and survived for more than 17 months, grade 1 hypertension and fatigue are the only side effects (CTCAE v5.0) [327].

The 2022 American Association for Cancer Research (AACR) Annual Meeting recently announced advances in CAR-T therapies for solid tumors (Fig. 3). The first phase 1/2 clinical trial results to enhance CAR-T cell therapies’ activity via a CLDN6-encoding mRNA vaccine (CARVac) were presented at the annual meeting. CLDN6 is a tumor-specific antigen that is considered an ideal target. The vaccine provides a stimulus to the adoptively transferred CAR-Ts by making dendritic cells express the target antigen of the CAR [328]. In the 6th week of this trial, 14 patients with relapsed or refractory advanced could participate in the efficacy evaluation. Six patients (4 patients with testicular cancer and two with OC) had PR, and the ORR was nearly 43% [329]. About 40% of patients experienced a CRS and have no neurotoxicity.

Intravenous or intraperitoneal injections of MUC16 CAR-T cells could inhibit OC or eliminate tumors in mouse models [330]. 80% of epithelial OC cells express MUC16, a tumor marker [331]. Therefore, MUC16 may be an ideal antigenic target in CAR-T cell therapies for OC.

L1-CAM is highly overexpressed in OC, while studies have demonstrated the antitumor activity of L1-CAM CAR- T cells in OC xenograft models [332].

In addition, in OC in vivo or in vitro studies, CAR therapies targeting FRα, HER2, uPAR, 5 T4, ALPPL2, B7H3, PTK7, TAG72, CD47, OR2H1, and CDH6 have been suggested as promising therapeutic approaches [195, 213, 306, 333–338] (Fig. 3). FRα, HER2, ALPPL2, B7H3, and TAG72 have entered OC clinical studies, but there are still no reports. We list here the NCT numbers of the most recently initiated or updated clinical trials for these targets: FRα (NCT03585764), HER2 (NCT04660929), ALPPL2 (NCT04627740), B7H3 (NCT04670068), TAG72 (NCT05225363). Clinical trials targeting MUC1 and CD70 have also been carried out (NCT05239143, NCT02830724), but there is little related preclinical evidence for OC treatment.

Several strategies are available to improve the antitumor effects of CAR therapies for OC. For example, CXCR1 is a G protein-coupled receptor with a high affinity for binding IL-8. IL-8 production is increased in a wide range of solid tumor malignancies. It has been shown that CXCR1 expressed on CAR-NK cells enhances migration and infiltration of CAR-T cells by matching chemokines secreted by tumors and, more importantly, enhances anti-tumor responses in vivo [339]. In an OC mouse model, SynNotch CAR T cells showed better control of tumor load [334]. In addition, Song et al. investigated the FRα-specific site scFv (MOv19) binding to CD137 (4-1BB) co-stimulatory pattern (MOv19-BBζ). MOv19-BBζ CAR-T cells were used in animal models of FRα + OC in intraperitoneal, subcutaneous, and metastatic pulmonary models with positive therapeutic effects [340]. But CD137 signaling does not increase the anti-tumor effect in vivo despite improved T cell persistence.

Targeting tumor neovascularization and extracellular matrix is also a strategy, and recent studies have shown that Annexin A2 (ANXA2) has been detected in OC. Overexpression of ANXA2 mediates extracellular matrix degradation and neointima formation through fibrinolytic enzyme production and is associated with invasion and metastasis [341]. Elimination of CSCs is considered a promising strategy. Markers present on the surface of CSCs, such as CD133, CD44, and CD47, may be targets for CAR. The antitumor effects of CAR targeting CD133, CD44, and CD47 have been validated in OC models [342]. Combining CAR intervention with other therapy has been tried by several studies. For example, studies have shown synergistic effects of paclitaxel with ErbB CAR -T cells in vivo [343].

### Glioma

As the most common primary tumors in the CNS, gliomas could be classified as low-grade gliomas or glioblastomas (GBM) based on histological and molecular features [344]. As one of the most malignant and recurrent solid tumors, GBM has a global incidence rate of 10/100000. Individuals with GBM have a five-year survival rate of fewer than 10% [345]. It has been established that patients with GBM have a poor prognosis after conventional therapy. As a promising therapeutic approach, CAR-T cell therapy has identified multiple specific targets that may address the challenge of treating glioma.

Up to now, well-studied CAR-T cell therapy targets in glioma include IL13Rα2, EGFRvIII, HER2, and GD2 (Fig. 3). IL13Rα2 is rarely expressed in normal brain cells but is highly expressed in GBM. This specificity makes them ideal targets for CAR-T cell therapy in GBM. The expression of IL13Rα2 in gliomas could also be used to assess prognosis [346]. In two phase 1 trial reported by Brown et al. in 2015 and 2016, several patients treated with IL13Rα2 CAR-T cells showed good tolerability (NCT02208362, NCT00730613) [347, 348]. Clinical trial (NCT02208362) reports clinical experience with one patient. After IL13Rα2 CAR T-cell treatment, regression of all intracranial and spinal tumors was observed. They observed therapeutic effects against GBM, with elevated immune cells and factor levels in the cerebrospinal fluid. This clinical response continued for 7.5 months after the initiation of CAR T-cell therapy [347]. In another clinical study (NCT00730613), three patients with recurrent GBM were treated with IL13Rα2-redirected CAR CD8+ T cells. Three patients with recurrent disease were well tolerated with controlled ephemeral brain inflammation. Transient evidence of antitumor response was observed in 2 of these patients [348]. Unfortunately, in this trial, one patient experienced shorter remission, which may be related to the loss of IL13Rα2 antigen on the relapsed tumor, leading to the poor response of IL13Rα2 CAR-T cells against GBM. In addition, the intraventricular infusion exhibited a better ability to eliminate distant tumor growth than intracranial tumor infusion. Recently, IL13Rα2 CAR-T has been shown to activate immune cells in vivo via IFN-γ-mediated pathways [349].

Tumor-specific antigens are relatively rare. EGFRvIII has received attention as a mutant of the EGFR receptor; it is expressed only on the surface of tumor cells but not in normal tissues (Fig. 3). In a phase 1 study, ten patients with recurrent GBM expressing EGFRvIII were treated with EGFRvIII CAR-T cells (NCT02209376). CAR-T cells exerted antitumor effects and mediated antigen deficiency and resistance in GBM. In these ten subjects, the median OS was 251 days (approximately 8 months), and no patients had severe adverse events [350].

HER2 has tyrosine kinase activity and belongs to the ErbB family; it could reportedly promote cell proliferation and further development of tumors [351] (Fig. 3). In a recent phase 1 clinical trial (NCT03500991), repeated topical administration of HER2 CAR-T cells to children and young people with recurrent or refractory CNS tumors, including diffuse midline gliomas, yielded no dose-limiting toxicity. High CXCL10 and CCL2 levels were detected in the cerebrospinal fluid [352]. This finding suggests CAR-T cell products with chemokine receptor expression may converge on CXCL10 and CCL2 expression sites to promote CAR-T cell binding to targets.

Another target that has been studied in depth is GD2 (Fig. 3). This disialoganglioside is usually expressed on peripheral neurons and parts of the CNS and acts as a promoter of intercellular adhesion [353]. It is highly and almost universally expressed in neuroblastoma tissue [354]. H3K27M+ diffuse intrinsic pontine glioma (DIPG) and spinal diffuse midline glioma (DMG) are highly aggressive, universally fatal tumors with few treatment options. A paper published in Nature reported the clinical experience of four patients with H3K27M mutated DIPG or spinal DMG who received intravenous infusion therapy with GD2 CAR-T cells [355]. Three patients demonstrated clinical and radiological improvement, and increased concentrations of inflammatory factors were found in cerebrospinal fluid and blood. Patients did not experience on-target, off-tumor toxicity. Observed improvements in neurological function highlight the potential of tumor cell-specific therapies in functional recovery [355].

Despite the advances in CAR-T cell therapy, patients that undergo single-targeted treatment are prone to relapse and subsequent resistance due to the molecular heterogeneity and evolution of tumors. Therefore, targeting multiple antigens or immunosuppressive cytokine antagonism is recommended [356]. In short, such combination therapies still require expanding the targeted antigen pool or developing new CAR-T cell types.

EphA2 is also overexpressed in GBM, enhancing tumorigenesis and migration. Lin et al. performed a phase 1 trial of EphA2 CAR-T cells in three EphA2-positive recurrent GBM patients with transient clinical efficacy and initial tolerability at the tested dose level (NCT03423992) (Fig. 3). One was SD (transit diminishment), and 2 were PD. No adverse events up to level 3 were reported [357].

B7H3, an immune checkpoint involved in tumor migration and invasion, has recently become a target of CAR-T cell therapy [358] (Fig. 3). Tang et al. reported using B7H3 CAR-T cells to treat a patient with recurrent GBM and showed that despite mediating a short-term antitumor response in situ, resistance quickly developed [359].

Moreover, NKG2DL is highly expressed in glioblastoma. One study substantiated synergy between NKG2DL CAR-T cells and radiation therapy in treating a mouse glioma model and the ability to lyse GBM tumor cell lines and CSCs (U-251 MG, T98G, U-87 MG, HTB185, GSC3) effectively [360, 361]. NKG2DL and B7H3 have also entered clinical studies to treat glioma (Fig. 3).

MUC1, CD147, and MMP2 have also been considered auspicious targeting sites, and related clinical trials are underway (Fig. 3). Here are the NCT numbers: MUC1 (NCT02617134, NCT02839954), CD147 (NCT04045847), and MMP2 (NCT04214392).

CSPG4 is also widely expressed in various malignancies [362]. CSPG4 CAR-T cells resisted brain tumor growth in cultured neurosphere and glioma xenograft mice models without signs of tumor escape [363].

A recent novel CAR-T cell, using the scorpion toxin peptide chlorotoxin (CLTX) as the targeting structural domain, has been reported to inhibit tumors in a xenograft GBM model with no observed off-target effects [364]. The pioneering design of the CLTX CAR-T suggests that designing CAR-T cells using tumor-binding peptides is promising and could potentially reduce tumor escape, further expanding the tumor-targeting antigen pool.

Besides, Podoplanin (PDPN) is a mucin-like glycoprotein whose overexpression has been associated with mesothelioma, EC, LCa, and mesenchymal GBM [365]. The third-generation PDPN-redirected CAR-Ts were reported to mediate efficient anti-tumor responses against PDPN-positive GBM cell lines (LN319, U87MG) in vitro and inhibit r growth in a glioma mouse xenograft model [366]. However, PDPN-redirected CAR-Ts were found with frequent toxicity in preclinical models by targeting normal tissue expressed PDPN. To solve this issue, a cancer-specific monoclonal antibody CasMab (LpMab-2), which only reacts with the aberrant tumor tissue expressed glycosylated PDPN, was developed [367].

Targeting the tumor stroma and vascular system embodies a novel strategy for tumor suppression. It is used to treat glioma.

It has been shown that p21-activated kinase 4 (PAK4) inhibition normalizes the tumor vascular microenvironment and makes GBM more sensitive to CAR-T cell therapy [368].

Moreover, P32 CAR-T cells have antitumor and anti-angiogenic effects in gliomas [369].

FAP has been reported in a recent study as an ideal immunotherapeutic antigen for targeting tumor cells and stroma [370].

Studies in animal models have indicated that the proteins CD133, AXL, c-mesenchymal-epithelialmesemesenchymal-epithelialnchymal-epithelial transition factor (c-MET), as well as factor-inducible 14 (Fn14) are specific targets for CAR-T cell treatment in GBM [371–373].

In conclusion, numerous TAA have significant potential therapeutic effects in GBM, and their applications need further development.

Targeted combinations of multiple tumor antigens mitigate tumor escape and enhance T-cell effects. For example, for the treatment of glioma, a tandem CAR-T cell functions more robustly and persistently and reduces antigen escape by crosslinking HER2 and IL13Rα2 receptors than a single targeted CAR-T cell [374]. In addition, a trivalent CAR-T cell combines three CAR molecules that could target HER2, IL13Rα2, and EphA2, respectively, and kill tumor cells expressing single or multiple antigens, expanding the therapeutic range [375]. The synNotch-CAR-T cells, which have become popular in recent years, could induce the expression of CARs by relying on a particular antigen (e.g., the GBM neoantigen EGFRvIII) to initiate the effect and kill based on a highly homogeneous antigen or set of antigens (e.g., EphA2 and IL13Rα2) [376]. This design allows CAR-T cells to display enhanced antitumor activity and persistence without causing extra-tumor killing. In a study, Lp2 CAR-T cells were designed to target PDPN-expressing glioma cells to exclude normal PDPN-expressing cells. Concurrent use of Lp2 CAR-T and third-generation recombinant herpes simplex virus-1 lytic virus G47Δ further inhibited tumor growth and improved survival [377].

Several different approaches are now proven to improve the safety and efficacy of CAR-T immunotherapy, including combination therapy between radiotherapy and CAR-T therapy and intracerebroventricular injection of CAR-T. These results provide new ideas for the future design of CAR-T in the treatment of solid tumors.

### Head and neck squamous cell carcinoma (HNSCC)

Approximately 890,000 new HNSCCs are diagnosed each year [378]. Conventional treatment for HNSCC is primary radiotherapy, but resistance may develop due to tumor heterogeneity [379]. CAR-T cell treatments have advanced considerably in HNSCC in current history. The following are the possible targets of CAR-T therapies for HNSCC observed so far.

CSPG4 interacts with α4β1 integrin to directly regulate cell adhesion. CSPG4 CAR-T cells have been shown to inhibit the growth of various solid tumors in preclinical studies, including BC, HNSCC, and mesothelioma [185].

MUC1, PD-L1, CD70, and CD44v6 have been identified as promising targets for CAR-T cells in the treatment of HNSCC and have been confirmed in preclinical studies [380–382] (Fig. 3).

Several optimization strategies have been developed to address the problems faced by CAR-T cell therapies in HNSCC (Fig. 3). Rosewell et al. established a construct encoding a PD-L1-blocking antibody and IL-12p70 binary lysing adenovirus (CAd), and local treatment combined with CAd12_PD-L1 and systemic HER2 CAR-T cell infusion in an HNSCC xenograft model improved survival to > 100 days [383]. This suggests that CAd12_PD-L1 enhances the antitumor effects of HER2 CAR-T cells. Another study designed a novel CD98- or EGFR- redirected UniCAR-T cell to lyse radioresistant HNSCC cells effectively, thus potentially improving the prognosis of radioresistant cancer patients [384]. To improve the affinity, cetuximab-constructed CAR-T cells were highly responsive to EGFR-positive HNSCC cells [385].

CAR-T cell clinical studies targeting HER2 (NCT03740256) and MUC1 (NCT05239143) for the treatment of HNSCC have been performed (NCT03356795).

### Other solid tumors

Most patients with thyroid cancer (TC) have a good prognosis, but metastatic and advanced TC have limited treatment options and a poor prognosis.

ICAM-1 plays a vital role in cell adhesion, cell signaling, and transendothelial migration of leukocytes to sites of inflammation [386] (Fig. 3). Irene et al. demonstrated that papillary TC and undifferentiated TC were associated with increased ICAM-1 [387]. ICAM-1 CAR-T cell showed substantial anti-tumor effects in TC cell lines (8505C, BCPAP, FRO, KHM-5 M) and xenograft mice models. They were also the first to report that ICAM-1 CAR-T cells could kill undifferentiated and hypofractionated TC cells in vitro and in vivo [387]. Interestingly, ICAM-1 CAR-T cells could induce increased ICAM-1 expression [388].

In addition, GFRα4 and TSHR are promising targets for treating TC and have been validated in preclinical models [389, 390] (Fig. 3).

Renal cell carcinoma (RCC) is currently one of the most malignant urological cancers [391]. CAR-T cell therapy has emerged as a new strategy for RCC treatment, but the current results are not satisfactory.

Cor H J Lamers et al. investigated the efficacy and safety of the first-generation CAIX CAR-T cells in 12 patients with carbonic anhydrase IX (CAIX)-positive metastatic renal cell carcinoma (mRCC) in a phase 1/2 trial (Fig. 3). Ultimately, no clinical responses were observed, and severe toxicity reactions occurred [392]. Nevertheless, the trial provided valuable lessons. A recent study has improved the design and composition of CAIX CAR-T cells. Their results suggest that CAIX BBζ CAR4/8 T cells are highly effective immunotherapy for RCC and promising for clinical application [393].

In addition, several preclinical studies have found that CAR cell therapies targeting L1-CAM, c-MET, PD-L1, EGFR, HER2, CD70, and B7H3 could suppress RCC [189, 320, 394–397]. CAR-T cells targeting EGFR, CAIX, CD70, B7H3, VEGFR-2, ROR2, MUC1, and GPC3 are currently in clinical trials to treat RCC (Fig. 3).

Cervical cancer (CC) is the fourth most prevalent malignancy and the fourth most significant cause of cancer mortality in women globally, according to a 2018 report [398]. Treatment with CAR-T cells for CC is in its infancy.

Preclinical research indicates that NKG2DL, MSLN, and PD-L1 are all excellent targets for CAR-T cells in the therapy of CC [399–401]. Furthermore, CAR-T cell clinical studies have been performed targeting MSLN, PD-L1, GD2, PSMA, and MUC1 (Fig. 3).

Osteosarcoma (OS) and Ewing’s sarcoma (EWS) have a poor prognosis once metastasis or recurrence occurs.

In a phase 1/2 clinical study, 19 HER2 positive subjects (16 OS, 1 EWS, one primitive neuroectodermal tumor, and one protofibroblastic small round cell tumor) were treated with HER2 CAR-T cells (Fig. 3). Four of 17 evaluable patients were stable at 12 weeks to 14 months, three had their tumors removed, and one had ≥90% tumor necrosis (NCT00902044). No patient experienced adverse T-cell infusion-related events except one patient who developed a fever on the highest dose [402].

CAR-T cells targeting IL-11Rα, GD2, EphA2, ROR1, type I insulin-like growth factor receptor (IGF1R), B7H3, CD166, VEGFR-2, and NKG2D have also been shown to have a therapeutic effect in OS and EWS models [403–411] (Fig. 3). These new findings give hope for the future of CAR-T immunotherapy for solid tumors.

Although CAR-T cells have more alternative targets in treating solid tumors and their applications are up-and-coming, they have shown limited antitumor activity in clinical trials. Accordingly, the use of CAR-T cell therapies among solid tumors still needs to be further explored. This failure is due to multiple factors, such as lack of specific targets, inefficient homing and penetration in solid tumors, and the inhibitory effect of TME on CAR-T cells.

CAR-T cell therapy targets commonly used in solid tumors are summarized (Fig. 3).

## Conclusions and outlooks

CAR-T cell therapy based on gene-editing technology has gained significant momentum in recent years, showing remarkable results in clinical applications and bringing a new dawn to immunotherapy for tumor patients.

Current evidence substantiates that CAR-T cell therapies are associated with excellent response rates in patients with hematologic malignancies [412]. Well-developed targets are available in B-ALL, B-NHL, and MM, and the FDA has approved four CAR-T products targeting CD19 and two targeting BCMA to treat these diseases. There are also targets in AML and HL that have good clinical outcomes, with CAR-T cell therapies targeting them. In T-ALL, CLL, and T-NHL, the therapeutic efficacy of CAR-T cell therapy has yet to be further improved, and suitable targets are still being explored [9]. The high heterogeneity of cancer and the tendency of malignant cells to recur through antigen-negative relapse mediated by antigen escape mechanisms under therapeutic pressure makes it difficult for CAR-T cell therapies targeting a single target to work well [9]. It should also be borne in mind that many targets are expressed in non-malignant tissues. This poor target specificity leads to severe consequences involving CAR-T cell attacks on normal hematopoietic tissues, such as T-cell self-mutilation caused by CAR-T cell therapies for T-cell tumors [5]. In addition, it has recently been found that in a mouse leukemia model, CAR-T cells could induce antigen transfer from cancer cells to T cells through a process called phagocytosis, resulting in antigen loss, reduced target density on tumor cells, and reduced effectiveness of therapy [413].

The solid tumor studied maturely mainly contains digestive system cancer, MEL, glioma, and so on. Treatment with CAR-T cells for CC and RCC is still in its infancy. Although research on CAR-T cell therapy has now shifted attention to solid tumors, studies on solid tumors continue to face difficulties due to multiple factors [414]. On the one hand, it is due to the characteristics of solid tumors. Solid tumor cells are heterogeneous, and few TSA has been found on the tumor cell’s surface. Therefore, targets are often selected for TAA that is highly expressed on the tumor surface and lowly expressed in normal tissues. Still, this approach may lead to off-target tumor toxicity, CRS, and antigen loss [415].

On the other hand, it is due to the complexity of the solid tumor microenvironment, which is highly abnormal with varying degrees of vascular collapse and dense rigid stromal structures [415]. These characteristics hinder the infiltration of CAR-T cells. In addition, there is a resistance of immunosuppressive cells and inhibitory factors in the tumor microenvironment [416]. The combination of the above factors resulted in difficult infiltration of CAR-T cells and aggravated the failure. To address the above problems, CAR-T modification and combination therapy are two good directions [417]. However, here we will still suggest possible directions for improvement based on the difficulties raised. First, we could enhance antigen-specific recognition of CAR-T cells and overcome the heterogeneity of tumor cell antigens. Second, targeting TME will be attractive, so improving TME infiltration of CAR-T cells and targeting immunosuppressive and metabolic tumor microenvironments would be helpful.

The manufacturing process of CAR-T cells may also be problematic. Circulating tumor cells are collected along with lymphocytes during collection. CAR could be transduced into these tumor cells, binding to targets expressed by the same cell, causing the tumor cells to escape [418]. The manufacture of CAR-T cells is also time-consuming and costly, and the use of allogeneic CAR-T cells from healthy donors, which are knocked out of HLA and endogenous TCR by CRISPR technology, seems to help us solve this problem [419].

**Fig. 4:**
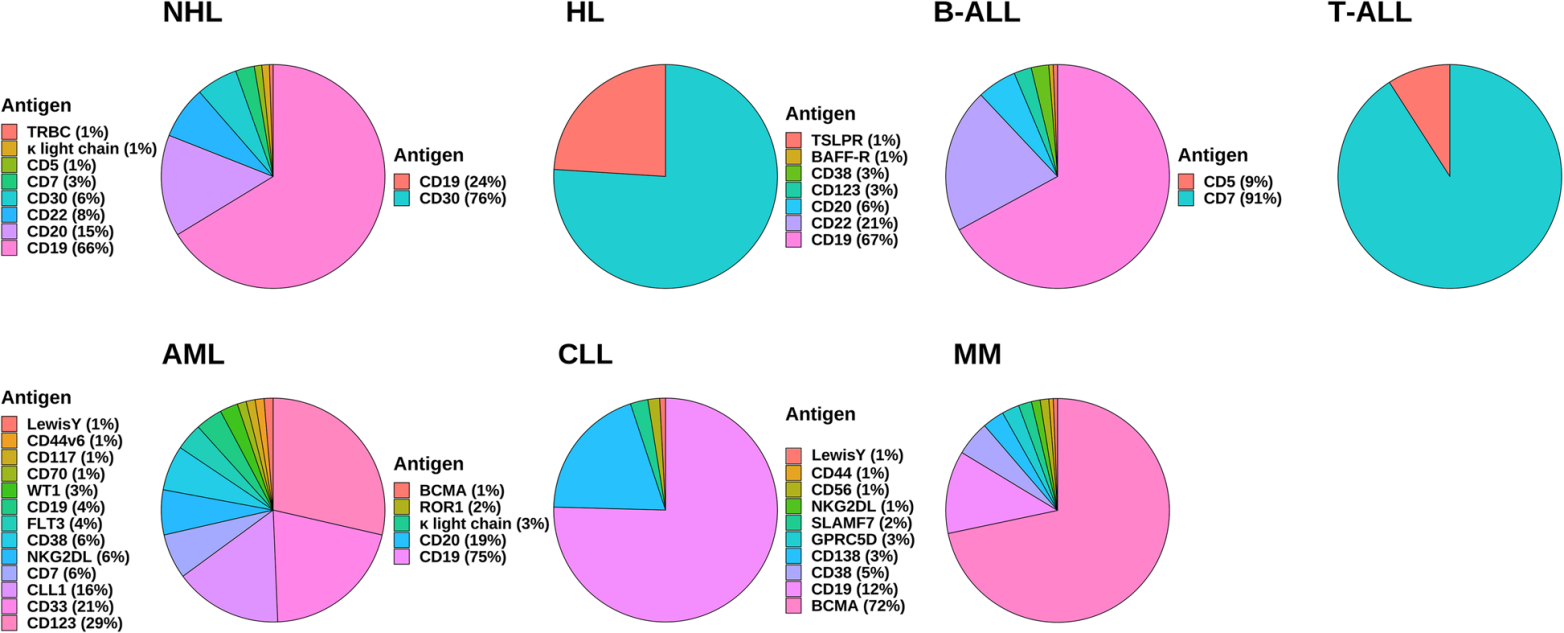
The proportions of clinical trials on CAR-T cell therapy targets in hematological malignancies. NHL: non-Hodgkin’s lymphoma; TRBC: T cell receptor β-chain constant domains; HL: Hodgkin’s lymphoma; B-ALL: B-acute lymphoblastic leukemia; BAFF-R: B-cell activating factor receptor; TSLPR: thymic stromal lymphopoietin receptor; T-ALL: T-acute lymphoblastic leukemia; AML: acute myeloid leukemia; WT1: wilms tumor 1; FLT3: FMS-like tyrosine kinase 3; NKG2DL: natural killer group 2 member D ligand; CLL1: C-type lectin like molecule 1; CLL: chronic lymphocytic leukemia; BCMA: B-cell maturation antigen; ROR: receptor tyrosine kinase like orphan receptor; MM: multiple myeloma; SLAMF7: signaling lymphocytic activation molecule F7; GPRC5D: G protein-coupled receptor class-C group-5 member-D

**Fig. 5:**
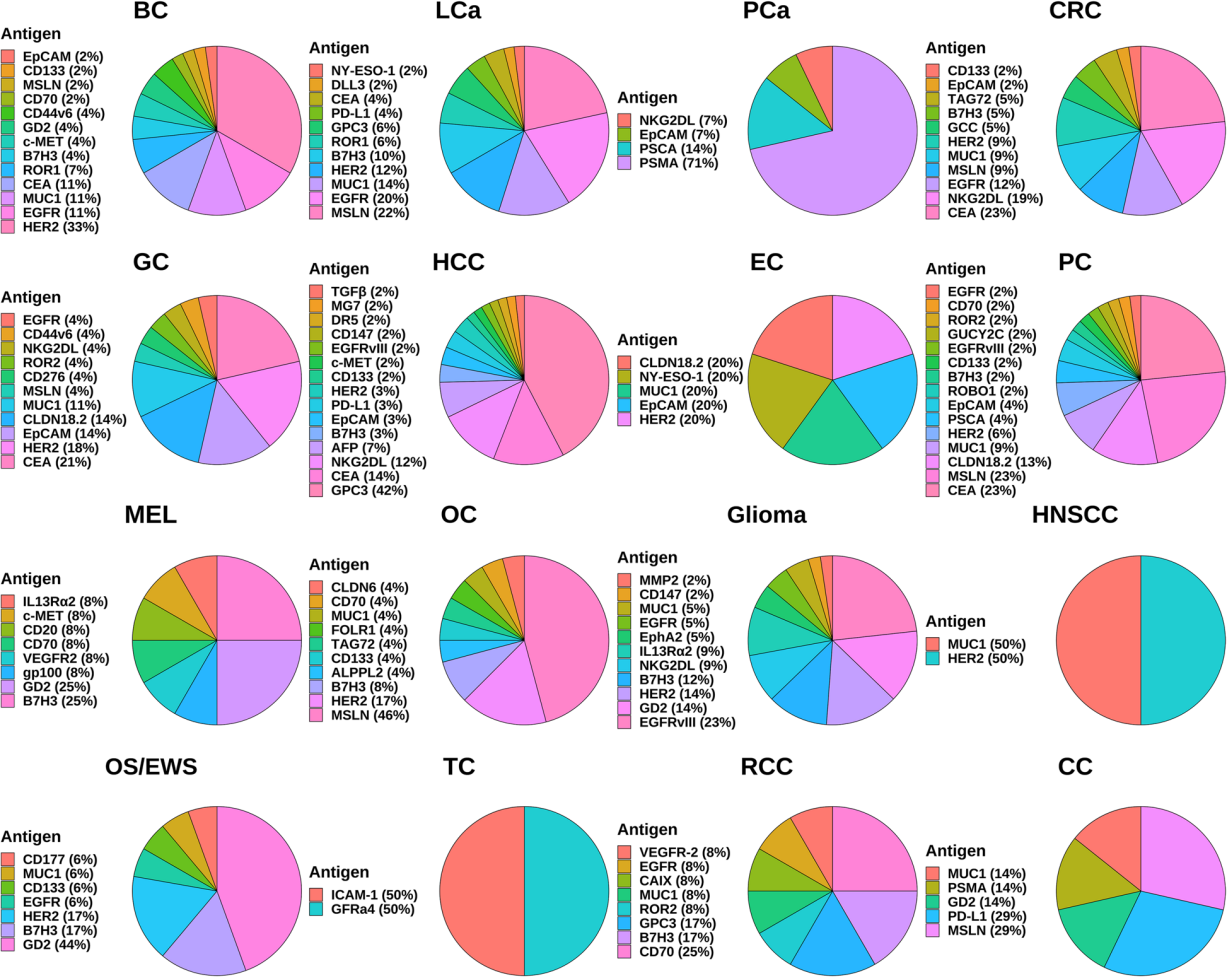
The proportions of clinical trials on CAR-T cell therapy targets in solid tumors. BC: breast cancer; MSLN: mesothelin; GD2: ganglioside2; ROR: receptor tyrosine kinase like orphan receptor; CEA: carcinoembryonic antigen; MUC1: mucin 1; EGFR: epidermal growth factor receptor; HER2: human epidermal growth factor receptor 2; LCa: lung cancer; NY-ESO-1: New York esophageal squamous cell carcinoma-1; DLL3: delta-like ligand 3; PD-L1: programmed death-ligand 1; PCa: prostate cancer; NKG2DL: natural killer group 2 member D ligand; PSCA: prostate stem cell antigen; PSMA: prostate-specific membrane antigen; CRC: colorectal cancer; GC: gastric cancer; CLDN: claudin; HCC: hepatocellular carcinoma; GPC3: glypican-3; EC: esophageal cancer; PC: pancreatic cancer; GUCY2C: Guanylyl cyclase C; MEL: melanoma; VEGFR-2: vascular endothelial growth factor receptor 2; OC: ovarian cancer; ALPPL2: alkaline phosphatase placental-like 2; HNSCC: head and neck squamous cell carcinoma; OS: osteosarcoma; EWS: Ewing’s sarcoma; TC: thyroid cancer; ICAM-1: intercellular adhesion molecule-1; RCC: renal cell carcinoma; CAIX: carbonic anhydrase IX; CC: cervical cancer

In the current situation, the rational selection and application of CAR-T cells targeting different targets is the most important guarantee of achieving good clinical results. Many CAR-T targets are being discovered, and target selection is critical (Table 1). Regarding the number of clinical trials corresponding to the different targets for each tumor (Figs. 4 and 5), CD19, CD20, and CD22 are generally considered essential targets for lymphoma and leukemia. In contrast, BCMA is usually considered an important and necessary target for multiple myeloma. EGFRvIII and GPC3 are crucial targets in the solcriticalsection in glioma and HCC, respectively. Besides, tumor-associated glycoforms of conventional antigens, including SLAMF7, CLEC14A, PDPN, PODXL, and CD44, could also be ideal target antigens. These have been described in detail in the text. The perfect target should be highly expressed uniformly on the surface of tumors at different stages, not expressed in normal tissues, not subject to specific therapeutic pressures that may lead to downregulation or elimination, and participate in the pathophysiology of the disease [18]. If the target does not meet these requirements, side effects like tumor antigen-negative relapse and extratemporal tissue toxicity may lead to toxicity.

**Table 1:** Target antigens of CAR-T cell therapy

Antigen	Function	Normal tissue expression	Malignant tissue expression	Progress	Application
CD19 (B lymphocyte surface antigen B4, T cell surface antigen LEU-12)	Assists in the differentiation of primitive B cells and maintain the balance of mature B cells in peripheral blood	Widely expressed in B cell lineages (from pre-B cell stage to plasma cell) and follicular dendritic cells	B-NHL, HL, B-ALL, CLL, MM	FDA approved (B-NHL, B-ALL); phase 3 (CLL); phase 2 (MM); phase1 (HL)	The most effective target of CAR-T therapy for B-ALL and B-NHL, the most well-studied CAR-T target for CLL therapy
CD22 (Siglec-2, BL-CAM, T cell surface antigen LEU-14)	Regulates the activation of B cells, plays an important role in maintaining B cell tolerance	Widely expressed in B cell lines (from pre-B cell stage to mature B cell)	B-NHL, T-NHL, B-ALL	Phase 2 (B-NHL, B-ALL)	Primary target for treatment after CD19 CAR-T relapsed B-ALL; CD19/CD22 bispecific CAR-T has good curative effect and reduces the risk of CRS due to CD19 CAR-T therapy
CD20 (B Lymphocyte surface antigens B1, Bp35, leU-16, MS4A1)	Regulates B cell activation and proliferation	Subsets of T cells and B cells	B-NHL, B-ALL, CLL, MEL	Phase 2 (B-NHL); phase 1/2 (B-ALL, CLL); early phase 1 (MEL)	Primary target for treatment after CD19 CAR-T relapsed CLL and B-NHL, CD19/CD20 dual target CAR-T is widely used in the treatment of B-NHL
κ light chain	Participates in the formation of immunoglobulin	B cells	B-NHL, CLL, MM	Phase 1 (B-NHL, CLL); preclinical (MM)	
λ light chain	Participates in the formation of immunoglobulin	B cells	B-NHL, CLL	Preclinical (B-NHL, CLL)	Alternative target for MCL therapy
CXCR5 (CD185)	Binding B cell chemokine BL participates in B cell migration	Mature B cells	B-NHL	Preclinical (B-NHL)	
CD5 (LEU1)	Regulates T - B cell interactions and interacts with CD72	Thymocytes, T and B cell subpopulations	T-NHL, T-ALL	Phase 1 (T-NHL, T-ALL)	
CD7 (LEU 9, GP40, TP41)	T cell costimulation and interacts with SECTM1	Thymocytes, T cells, natural killer cells and pluripotent hematopoietic stem cells	T-NHL, T-ALL, AML	Phase 1/2 (T-ALL, AML); phase 1 (T-NHL)	The primary target of CAR-T therapy for T-ALL
TRBC	Participates in the composition of TCR	T cells	T-NHL	Phase 1/2 (T-NHL)	
CD30 (TNF receptor superfamily member 8, TNFRSF8, Ki-1 antigen)	Regulates lymphocyte proliferation and cell death	Activated T and B cells, monocytes, and activated natural killer cells	T-NHL, HL	Phase 2 (T-NHL, HL)	The most viable CAR-T targets for HL therapy
CD4 (OKT4)	Activate T cells and participate in thymus differentiation	Thymocyte subsets, helper T cells, regulatory T cells, monocytes, and macrophages	T-NHL	Preclinical (T-NHL)	
CD38 (ADP ribocyclase 1)	Regulates cell activation, proliferation and adhesion	High levels of expression in plasma cells, early T cells and B cells, activated T cells, and germinal center B cells	B-ALL, AML, MM	Phase 1/2 (B-ALL, AML); phase 0 (MM)	BCMA/CD38 dual target CAR-T has shown good efficacy in MM treatment
CD123 (IL3RA)	Helps hematopoietic cells to proliferate and differentiate	HSC, dendritic cells, monocytes, endothelial cells	B-ALL, AML	Phase2/3 (AML); phase 1/2 (B-ALL);	The most studied CAR-T targets in AML therapy
BAFF-R (TNFRSF13C, CD268)	Promotes the survival of mature B-cells and the B-cell response	Highly expressed in spleen and lymph nodes and resting B cells, low expression in activated B cells, quiescent CD4+ T cells, thymus and peripheral leukocytes	B-ALL	Phase1 (B-ALL)	
TSLPR (CRL2, CRLF2)	Promotes the proliferation and differentiation of immune cells	Dendritic cells and CD4+ T cells	B-ALL	Phase 1/2 (B-ALL)	Targeting high-risk variants ALL
CD72	B cell activation and proliferation	B (not plasma) cells, mac, FDC, endo cells	B-ALL	Preclinical (B-ALL)	Targeted MLLr ALL
NKG2DL	Binding to NKG2D on NK cells induces cell-mediated cytotoxicity and destroys target cells	Not reported	T-ALL, AML, MM, Glioma, HCC, BC, GC, CRC, PCa, OS, EWS	Phase 1/2 (AML); phase 1 (Glioma, HCC, BC, GC, CRC, PCa); preclinical (T-ALL, MM, OS, EWS)	
CD33 (Siglec-3)	Participates in inhibiting proliferation of normal and leukemia bone marrow cells; lectin acitivity; adhesion	Monocytes, granulocytes, mast cells, and myeloid progenitors	AML	Phase 1/2 (AML)	
CLL1 (CLEC12A)	Modulates immune homeostasis	Not reported	AML	Phase 2/3 (AML)	
LewisY	May be involved in cell adhesion	Epithelial cells	AML, MM	Phase 1 (AML); preclinical (MM)	
FLT3 (CD135, FLK2)	Promotes the growth and differentiation of primitive hematopoietic cells	Polypotent bone marrow monocytes and pro-B cell precursors	AML	Phase 1/2 (AML)	For the treatment of FLT3+ AML with poor prognosis with conventional therapy
CD117 (c-kit, KIT)	Regulates gonad and pigment stem cell development	HSC	AML, OS, EWS	Phase 0 (AML); phase 1/2 (OS, EWS)	
Siglec-6 (CD327, OB-BP1)	Mediates sialic acid - dependent cell binding	B cells, placental trophoblast cells and granulocytes	AML, CLL	Preclinical (AML, CLL)	
CD70 (CD27L)	InducesT cell proliferation after costimulation, promotes the production of cytotoxic T cells and contributes to T cell activation and participates in the regulation of B cell activation, cytotoxicity of natural killer cells and immunoglobulin synthesis	Activated B cells and T cells, macrophages	AML, MEL, BC, HNSCC, EC, PC, OC	Phase 1/2 (BC, PC, OC); phase 1 (MEL); early phase 1 (AML); preclinical (HNSCC, EC)	
MPL (MPF, SMRP)	Regulates hematopoietic stem cell self-renewal, megakaryocyte and platelet development	Hematopoietic progenitor cells, megakaryocytes, platelets, endothelial cells	AML	Preclinical (AML)	
LILRB4 (CD85, LILRB3, ILT5, LIR3)	Participates in cytotoxicity mediated by natural killer cells	Monocytes, macrophages, bone marrow cells, partial T cells, natural killer cells, basophils, eosinophils, and B cells	AML	Preclinical (AML)	Targeting monocytic AML, which is common in children
Tim3 (CD366, HAVCR2)	Inhibits immune cell activation	Bladder	AML	Preclinical (AML)	
FRβ	Folate uptake	Not reported	AML, LCa	Preclinical (AML, LCa)	
CD44v6 (Epican, HUTCH-I, LHR, ECMR-III)	Participates in leukocyte rolling, homing and aggregation, enables leukocytes to adhere to endothelial cells, stromal cells and extracellular matrix	Most lymphohematopoietic cells	AML, MM, LCa, BC, GC, HNSCC	Phase 1/2 (AML, BC, GC, MM); preclinical (LCa, HNSCC)	
WT1 (WIT-1)	As a transcription factor involved in organ development, differentiation, proliferation and apoptosis	Spleen, kidney, heart, lung, prostate	AML	Phase 1 (AML)	
PR1	HLA-A2 restriction peptide	Not reported	AML	Preclinical (AML)	
MSLN	May help cells resist apoptosis	Mesothelial cells	AML, LCa, BC, GC, PC, OC, CRC	Phase 1/2 (LCa, BC, GC, PC, OC, CRC); preclinical (AML)	
BCMA (TNFRSF17, CD269)	Enables NFkB and JNK, promotes B cell survival and participates in humoral immunity regulation	Mature B cells	CLL, MM	FDA approved (MM); phase 0 (CLL)	The primary target of CAR-T therapy for MM
CD32b (FcγRII, FCGR2A, FCGR2B)	Regulates B cell function, induces phagocytosis and release of media	B cells, monocytes, granulocytes, platelets, and endothelial cells	CLL	Preclinical (CLL)	
FcμR	In combination with immunoglobulin M	Rarely expressed in healthy B cells or other hematopoietic cells	CLL	Preclinical (CLL)	
ROR1 (NTRKR1)	Resists cell apoptosis, activates cell migration and proliferation	Parathyroid glands, esophagus, stomach and duodenum	CLL, LCa, BC, OS, EWS	Phase 1 (CLL, LCa, BC); preclinical (OS, EWS)	
CD23 (Low affinity immunoglobulin, εFc receptor, BLAST-2, FcεRII, FCER2, CLEC4J)	Binds IgE, CD19, CD21, and CD81, controls B cell activation, and regulates of IgE	Mature B cells, activated macrophages, eosinophils, follicular dendritic cells, and platelets	CLL	Preclinical (CLL)	
SLAMF7 (CS1, CD319, CRACC)	Mediates the activation of natural killer cells and may be involved in lymphocyte adhesion	Natural killer cells and activated B cells	MM	Phase 1/2 (MM)	
SLAMF3 (CD229, Ly9)	Participates in the adhesion between T cells and helper cells	T and B cells	MM	Preclinical (MM)	
GPRC5D	Unknown	Folliculus pili	MM	Phase 1 (MM)	
NY-ESO-1 (CTAG1B, ESO1, LAGE2)	May be involved in cell cycle progression	Germ cells and placental cells	MM, MEL, LCa, EC	Phase 1/2(EC); Phase 1(LCa); Preclinical (MM, MEL)	
CD126 (IL6R, gp80)	Combined with IL6	Activated B and plasma cells, T cells, monocytes, epithelial cells, and fibroblasts	MM, MEL, PCa	Preclinical (MM, MEL, PCa)	
CD138 (Syndecan, SDC)	Participates in cell adhesion	Plasma cells, prob. cells, epithelial cells, nerve cells, and breast cancer cells	MM	Phase 1/2 (MM)	
CD56 (NCAM1)	Participates in homophilic and heterophilic adhesion	Neural tissue, natural killer cells, T cell subsets	MM	Phase 1/2 (MM)	
HER2 (ERBB2)	Inhibits apoptosis and promotes proliferation; promotes tumor angiogenesis and lymphatic vessel neogenesis; tyrosine kinase function	Low levels in very few tissues in adulthood	BC, Glioma, MEL, LCa, GC, PC, CRC, OC, HCC, HNSCC, EC, OS, EWS	Phase 2 (BC); phase 1/2 (Glioma, LCa, GC, PC, CRC, OC, OS, EWS); phase 1 (MEL, HNSCC, EC); preclinical (HCC)	Targets for CAR-T cell therapies for multiple solid tumors
EGFR(ERBB1)	Tyrosine kinase-type receptors, plays an important role in physiological processes such as cell growth, proliferation and differentiation	Mammalian epithelial cells, fibroblasts, glial cells, keratinocytes, etc.	BC, LCa, CRC, GC, PC, HNSCC, OS, EWS	Phase 2 (LCa); phase 1/2 (CRC); phase 1 (BC, GC, HNSCC, OS, EWS); preclinical (PC)	Target for CAR-T cell therapies for multiple solid tumors
c-MET (HGFR)	Receptor tyrosine kinase that regulates proliferation, scattering, morphogenesis and survival	Liver, brain，gastrointestinal tract, thyroid and kidney	BC, Glioma, HCC, LCa, CRC, GC	Phase 1 (BC); early phase 1 (HCC); preclinical (Glioma, LCa, CRC, GC)	
AXL (UFO, ARK)	A receptor tyrosine kinase (RTK), activate downstream signaling pathways such as AKT, MAPK, PI3K, mTOR, and NFκB pathways	Mammary gland	BC, Glioma	Phase 1 (BC); preclinical (Glioma)	
MUC1 (PEM, PUM, DF3, MAM-6)	Cell adhesion, regulation of signaling in the ERK, SRC and NF-κB pathways	The apical surface of epithelial cells, especially of airway passages, breast and uterus，some activated and unactivated T-cells.	BC, HCC, Glioma, GC, LCa, PC, CRC, HNSCC, OS, EWS, EC, OC, RCC, CC	Phase 1/2 (HCC, Glioma, BC, GC, LCa, PC, CRC, OS, EWS, EC, CC); phase 1 (HNSCC, OC)	A target for CAR-T cell therapies for multiple solid tumors
CD133 (Prominin-1, PROM1, AC133)	Participates in the regulation of MAPK and Akt signaling pathways	HSC, epithelial cells, endothelial cells and nerve cell precursors	BC, HCC, Glioma, LCa, PC, CRC, OC, OS, EWS, GC	Phase 1/2 (BC, PC, CRC, OC, OS, EWS); phase 1 (Glioma); preclinical (HCC, LCa, GC)	
EpCAM (CD326, TACSTD1)	Binds LAIR-1 and 2 to inhibit cellular activation and inflammation	Epithelial cells	BC, HCC, GC, CRC, PCa, PC	Phase 1/2 (PCa, HCC, GC, PC); phase 1 (BC, CRC)	
CSPG4 (NG2, MCSP, MCSPG, MSK16, HMW-MAA, MEL-CSPG)	Coordinates cell proliferation, adhesion, migration and survival	Perivascular cells, articular chondrocytes, and microglia in the central nervous system	BC, Glioma, MEL, HNSCC	Preclinical (Glioma, MEL, BC, HNSCC)	A target for the treatment of relapse of mixed lineage leukemia rearrangement (MLL-r)
ICAM-1 (CD54)	Regulates T cell activation	Endo, mono, hematopoietic and non-hematopoietic cells	BC, TC, GC	Phase 1 (TC); preclinical (BC, GC)	
TEM8 (ATR, GAPO)	Cell attachment and migration.	Umbilical vein endothelial cells	BC	Preclinical (BC)	
TROP2 (TACSTD2, GA733–1, M1S1)	Function as a growth factor receptor	Epithelial cells	BC, GC, PC	Preclinical (BC, GC, PC)	
FRα (FOLR1)	FOLR1 bound to folate is internalized into the nucleus and participates in cell proliferation and activation as a transcription factor	Kidney, lung and cerebellum; tissues of epithelial origin	BC, LCa, OC, GC	Phase 1 (OC); preclinical (LCa, BC, GC)	
EphA10	Receptor for members of the ephrin-A family， Binds to EFNA3, EFNA4 and EFNA5	TesticlesB7	BC	Preclinical (BC)	
PD-L1 (CD274, B7-H1)	Co-stimulation; inhibition	T cells, B cells, NK cells, DC,	BC, MEL, HNSCC, LCa, HCC, GC	Phase 1 (LCa); early phase 1 (HCC); preclinical (MEL, HNSCC, GC, BC)	Suppressive immune checkpoints
		Macrophages, Epithelial Cell			
GD2	Enhanced cell proliferation, motility, migration, adhesion and invasion	Peripheral neurons and part of the central nervous system	BC, Glioma, MEL, LCa, OS, EWS	Phase 1/2 (Glioma, LCa, BC); phase 1 (MEL); phase 1/2 (OS, EWS)	H3K27M mutation DIPG or spinal DMG
PTK7 (CCK4)	Inactive tyrosine kinase involved in Wnt signaling pathway; Functions in cell adhesion, cell migration, cell polarity, proliferation, actin cytoskeleton reorganization and apoptosis, has a role in embryogenesis, epithelial tissue organization and angiogenesis	Embryogenesis; some immature CD4+ recent thymus migration and plasma cell like dendritic cells, as well as low-level expression in some adult tissues	BC, LCa, OC	Preclinical (LCa, OC, BC)	
CEA (CEACAM5)	Structural protein of cell membrane; serum markers	Embryonic tissue	BC, HCC, LCa, GC, PC, CRC, PCa	Phase 2/3 (HCC, PC); phase 1/2 (LCa, BC, GC, CRC); preclinical (PCa)	Serum markers
gp100 (PMEL)	Plays a central role in the biogenesis of melanosomes	Mammary epithelium, sweat glands	BC, MEL, HCC	Early phase 1 (MEL); preclinical (BC, HCC)	
B7H3 (CD276)	Immune checkpoint molecules and co-stimulatory/co-inhibitory immunomodulatory proteins that play dual roles in the immune system.	Liver, lungs, bladder, testes, prostate, breast, placenta and lymphatic organs	LCa, Glioma, MEL, PC, PCa, HCC, EC, GC, OS, EWS, OC, CRC	Phase 1/2 (Glioma, PC, HCC, OC); phase 1 (MEL, LCa); early phase 1 (GC); preclinical (PCa, EC); Phase 1 (OS, EWS, CRC)	
DLL3 (SCDO1)	Binds to different Notch receptors and plays multiple functions in cell proliferation, differentiation and apoptosis	Low expression in most tissues	LCa	Phase 1 (LCa)	SCLC
MAGE-A1 (Cancer-testis Antigen)	Involved in transcriptional regulation through interaction with SNW1 and recruiting histone deactelyase HDAC1; inhibit notch intracellular domain (NICD) transactivation; play a role in embryonal development and tumor transformation or aspects of tumor progression	Germ cells of the testis, eggs and trophoblast cells of the placenta	LCa	Preclinical (LCa)	
GPC3 (DGSX, OCI5, SDYS, SGB, SGBS1, MXR7)	Alters Wnt/β-catenin, Hedgehog and FGF signaling to drive cell growth and inhibit differentiation	Placenta, fetal liver, fetal lung and fetal kidney, minimally expressed in most adult tissues	LCa, HCC, RCC	Phase 1/2 (HCC, LCa), phase 1 (RCC)	Target sites for the most commonly used CAR-T cell therapies for HCC; ediatric solid embryonal tumors
OR2H1	Interact with odorant molecules in the nose, to initiate a neuronal response that triggers the perception of a smell	Testis	LCa, OC	Preclinical (LCa, OC)	
CD47 (MER6, IAP)	Cell adhesion	Hematopoietic cells, epithelial cells, endothelial cells, mesenchymal cells	LCa, OC	Preclinical (OC, LCa)	
CD147 (BSG)	Cell adhesion; T cell activation	Leukocyte, erythrocyte, platelet, endothelial cells	LCa, HCC, Glioma	Phase 1 (HCC); early phase (Glima); preclinical (LCa)	
PSCA	Regulates cell proliferation	Prostate, bladder, placenta, stomach, colon and kidney	LCa, PC, CRC, GC	Phase 1/2 (PC); phase 1 (LCa, CRC, GC)	
FAP (SIMP, DPPIV, FAPA)	Cell surface glycoprotein serine protease, involved in extracellular matrix degradation and involved in many cellular processes, including tissue remodeling, fibrosis, wound healing, inflammation	Fibroblasts	LCa, Glioma, PC	Preclinical (Glioma, PC, LCa)	Targeting the tumor stroma
EphA2 (ECK, CTPA, ARCC2, CTPP1, CTRCT6)	Receptor tyrosine kinase; regulation of cell migration, integrin-mediated adhesion, proliferation and differentiation	Most epithelialthelial cells	LCa, Glioma, OS, EWS	Phase 1/2 (Glioma); preclinical (LCa, OS, EWS)	
CLEC14A (EGFR5)	Mediates intercellular adhesion	Not reported	LCa	Preclinical (LCa)	
CXCR4 (CD184, Fusin, NPYR, HSY3RR, LAP3, LCR1)	Receptor for chemokine stromal cell-derived factor-1 (CXCL12), chemotactic lymphocytes, involved in multiple physiological mechanisms in the body	Most tissues and organs	LCa	Preclinical (LCa)	Directing CAR-T cells to chemokine receptors
PSMA (GIG27, FOLH, NAALAD1, PSM)	Glutamate carboxypeptidase II enzyme	Expressed mainly in healthy prostate and to a lesser extent in other tissues, including intestine, brain, kidney, lacrimal gland and salivary gland	PCa	Phase 2 (PCa)	Important targets for CAR-T cells in prostate cancer
GUCY2C (GUC2C, STAR)	Guanylyl cyclase that catalyzes synthesis of cyclic GMP from GTP	Intestinal epithelium from the duodenum to the rectum	CRC, PC	Early phase 1 (PC); preclinical (CRC)	
CDH17	Cell adhesion	In the gastrointestinal tract and pancreatic duct	CRC, GC	Preclinical (GC, CRC)	
TAG 72 (CA72–4)	Has mucin properties	Not reported	CRC, GC, OC	Phase 1(CRC, OC), preclinical (GC)	
ROR2	Developmental regulatory proteins	Not reported	GC, PC, RCC	Phase 1/2 (RCC); phase 1 (GC, PC)	
CLDN18.2 (CLDN18, Claudin 18, SFTA5)	Plays a major role in tight junction-specific obliteration of the intercellular space, through calcium-independent cell-adhesion activity	Low-level expression in gastric mucosa differentiated epithelial cells	GC, PC, EC	Phase 1/2 (GC, PC, EC)	Common target in GC
B7H6	Triggers NCR3-dependent natural killer cell activation	Not reported	GC	Preclinical (GC)	
ARP2/3 (p41-ARC)	Regulates intracellular motility of the actin cytoskeleton, lysosomes, endosomes and mitochondria, migration	Multiple systems	GC	Preclinical (GC)	
NRP-1 (VEGF165R, NRP)	Regulation of angiogenesis, neuronal development and immune response	In adult tissues, it is highly expressed in heart and placenta; moderately in lung, liver, skeletal muscle, kidney and pancreas; and low in adult brain	GC	Preclinical (GC)	
DSC2	Component of intercellular desmosome junctions，involved in the interaction of plaque proteins and intermediate filaments mediating cell-cell adhesion， contribute to epidermal cell positioning (stratification)	Mainly skin, heart and esophagus	GC	Preclinical (GC)	
AE1/2	Mediate Cl−/HCO3- exchange, regulates intracellular pH, chloride concentration and bicarbonate metabolism	AE1 is mainly expressed on erythrocytes, AE2 is present in most tissues	GC	Preclinical (GC)	
CA19–9 (sialyl-Lewis)	Influence the initiation of apoptosis in activated T cells	Epithelial tissues of many organs, including the stomach	GC	Early phase 1 (GC)	
uPAR (CD87)	Acts as a receptor for urokinase plasminogen activator; plays a role in localizing and promoting plasmin formation	Brain	GC, OC	Preclinical (GC, OC)	
DR5	Induction of apoptosis	Expression on few tissues	HCC	Phase 1/2 (HCC)	
MG7	Unknown	Stomach	HCC	Preclinical (HCC)	
TGFβ (IBDIMDE)	Regulates cell growth and differentiation; induce epithelial mesenchymal transition and cell migration	Activated T cell or B cell;	HCC	Phase 1 (HCC)	
AFP	Transport function, bidirectional regulation as a growth regulator, immunosuppression, T lymphocyte-induced apoptosis; serum markers	Fetal hepatocytes, yolk sac	HCC	Phase 1 (HCC)	Serum markers
EGFRvIII	Unknown	Not reported	HCC, Glioma, PC	Phase 1/2 (Glioma, HCC, PC)	Optimal target in Glioma
CD44 (Hermes, Pgp1, H-CAM, Hutch)	Regulates cell proliferation, survival, etc.	A variety of tissues	HCC, OC	Preclinical (HCC, OC)	
TSPAN8	Cell adhesion, motility, proliferation, differentiation and immune cell function	Digestive System	PC	Preclinical (PC)	
ROBO1 (DUTT1)	Plays an important role in neurogenesis and immune response	Widely expressed, with exception of kidney	PC	Preclinical (PC)	
CD318 (CDCP 1)	May be involved in cell adhesion and cell matrix association; potential role in the regulation of anchorage versus migration or proliferation versus differentiation via its phosphorylation; possible marker for leukemia diagnosis and for immature hematopoietic stem cell subsets; belongs to the tetraspanin web involved in tumor progression and metastasis	Hematopoietic stem cells, epithelial cells	PC	Preclinical (PC)	
PODXL (PCLP, PCLP1, Gp200)	Plays an important role in the development and function of the glomerular filtration barrier	Kidney podocytes, megakaryocytes, vascular endothelial cells, and platelets	PC	Preclinical (PC)	
CD16 (FCG3, FCGR3, IGFR3, IMD20)	Low affinity Fc receptor; mediates phagocytosis and ADCC	Neuron, NK cells, Macrophages	MEL	Preclinical (MEL)	
αvβ3 (CD51, CD61, GP3A)	Regulates endothelial cell adhesion, migration, proliferation and apoptosis	Low expression in most tissues	MEL	Preclinical (MEL)	
VEGFR-2 (FLK-1)	Regulates lymphatic endothelial cells and vascular endothelial cells, the production of lymphatic vessels and blood vessels, the migration of lymphocytes	Endothelium of blood vessels, lymphatic vessels	MEL, RCC	Phase 1/2 (MEL, RCC)	Targeting the tumor vascular system
TRP1/2	Plays an important role in melanin synthesis	Melanocytes	MEL	Preclinical (MEL)	
GD3	Associated with cell growth, differentiation, malignant transformation, invasion and immunosuppression	Low expression in retinal pigment cells, central nervous system and normal melanocytes	MEL	Preclinical (MEL)	
IL13Rα2 (CD213A2, IL13BP, CT19)	High affinity membrane receptor for the anti-inflammatory cytokine interleukin 13 (IL-13)	Cancer germline antigens expressed in the testis, rarely expressed in normal brain cells	MEL, Glioma	Phase 1 (Glioma); preclinical (MEL)	The ideal target for the treatment of Glioma
CLDN6 (Skullin)	As an important tight junction protein involved in osmoregulation and barrier formation	High expression in fetal tissues such as stomach, pancreas, lung and kidney	OC, Testicular cancer	Phase 1 (OC, Testicular cancer)	
MUC16 (CA125)	Provides a protective lubricating barrier for particles and infection factors on mucosal surfaces	Corneal and conjunctival epithelia	OC	Phase 1 (OC)	
L1-CAM (CD171, HSAS, MASA)	A crucial role in neuronal cell adhesion and migration	Central nervous system	OC	Preclinical (OC)	
5 T4 (TPBG)	An inhibitor of Wnt/beta-catenin signaling by indirectly interacting with LRP6	Not expressed in most somatic adult human tissues	OC	Preclinical (OC)	
ALPPL2 (PLAP-like)	Alkaline phosphatase that could hydrolyze various phosphate compounds	Trace amounts in the testis and thymus, and in elevated amounts in germ cell tumors	OC	Preclinical (OC)	
CDH6	Cell adhesion	Highly expressed in brain, cerebellum, and kidney. Lung, pancreas, and gastric mucosa show a weak expression.	OC	Preclinical (OC)	
CXCR1	Binds IL-8 with high affinity	A variety of tissues	OC	Preclinical (OC)	
ANXA2 (ANX2L4, CAL1H, LIP2, LPC2, LPC2D, P36)	Mediation of extracellular matrix degradation and neointima formation through fibrinolytic enzyme production	Not reported	OC	Preclinical (OC)	
CLTX	Unkhown	Not reported	Glioma	Preclinical (Glioma)	Triggered an effective cytotoxic response also in tumor cells that do not or barely express other targetable antigens (IL13Rα2, HER2 and EGFR)
PDPN (GP36)	Induces platelet aggregation	Lymphatic endothelial cells	Glioma	Preclinical (Glioma)	
PAK4	Regulates cell adhesion, proliferation, migration, etc.	A variety of tissues	Glioma	Preclinical (Glioma)	
P32	Participates in several cellular signaling pathways involved in mitochondrial metabolism and dynamics, apoptosis, splicing, immune response, inflammation, and regulation	Mitochondria; syncytial trophectoderm, underlying cytotrophoblast, vascular endothelium, and in a subset of cells in the villi matrix of early and term placentas	Glioma	Preclinical (Glioma)	Dual anti-tumor and anti-angiogenic effects in glioma
Fn14	Cell surface receptor for tumor necrosis factor-associated weak apoptosis inducer	Low level expression in normal brain tissue	Glioma	Preclinical (Glioma)	
GFRα4 (GDNF family receptor alpha-4)	Mediates the GDNF-induced autophosphorylation and activation of the RET receptor	Thyroid gland	TC	Phase 1 (TC)	
TSHR (LGR3, CHNG1)	Receptor for the thyroid-stimulating hormone (TSH) or thyrotropin	Thyroid cells	TC	Preclinical (TC)	
CAIX	Catalyzes the interconversion between carbon dioxide and water and the dissociated ions of carbonic acid	Expression is restricted to very few normal tissues and the most abundant expression is found in the epithelial cells of gastric mucosa.	RCC	Phase 1 (RCC)	Common target in RCC
IGF1R (CD221, JTK13, MGC18216)	Receptor tyrosine kinase that mediates the action of insulin-like growth factor 1 (IGF1)	A variety of tissues	OS, EWS	Preclinical (OS, EWS)	
CD166 (ALCAM)	Adhesion; T cell activation	Activated T and B cells, NK cells, monocyte, epithelial cells, fibroblasts neurons, mesenchymal stem / progenitor cells	OS, EWS	Preclinical (OS, EWS)	
CD177 (NB1 GP, PRV-1)	Unknown	Neuron, Basophil, NK cells, T cell subset, monocyte, endothelial cells	OS, EWS	Preclinical (OS, EWS)

We should not stop the exploration of new targets. Proteomics, immunopeptidomics, and other techniques could search for new targets. The applicability of new targets to CAR-T cells should be evaluated mainly from stability, specificity, and pathophysiology. This is particularly important for the clinical application of CAR-T cells [18]. In preclinical experiments, some advanced technologies could also help us predict the effect of treatment, such as the tumor three-dimensional organoid model, which could well simulate the structural and functional heterogeneity of primary cells [420].

**Fig. 6:**
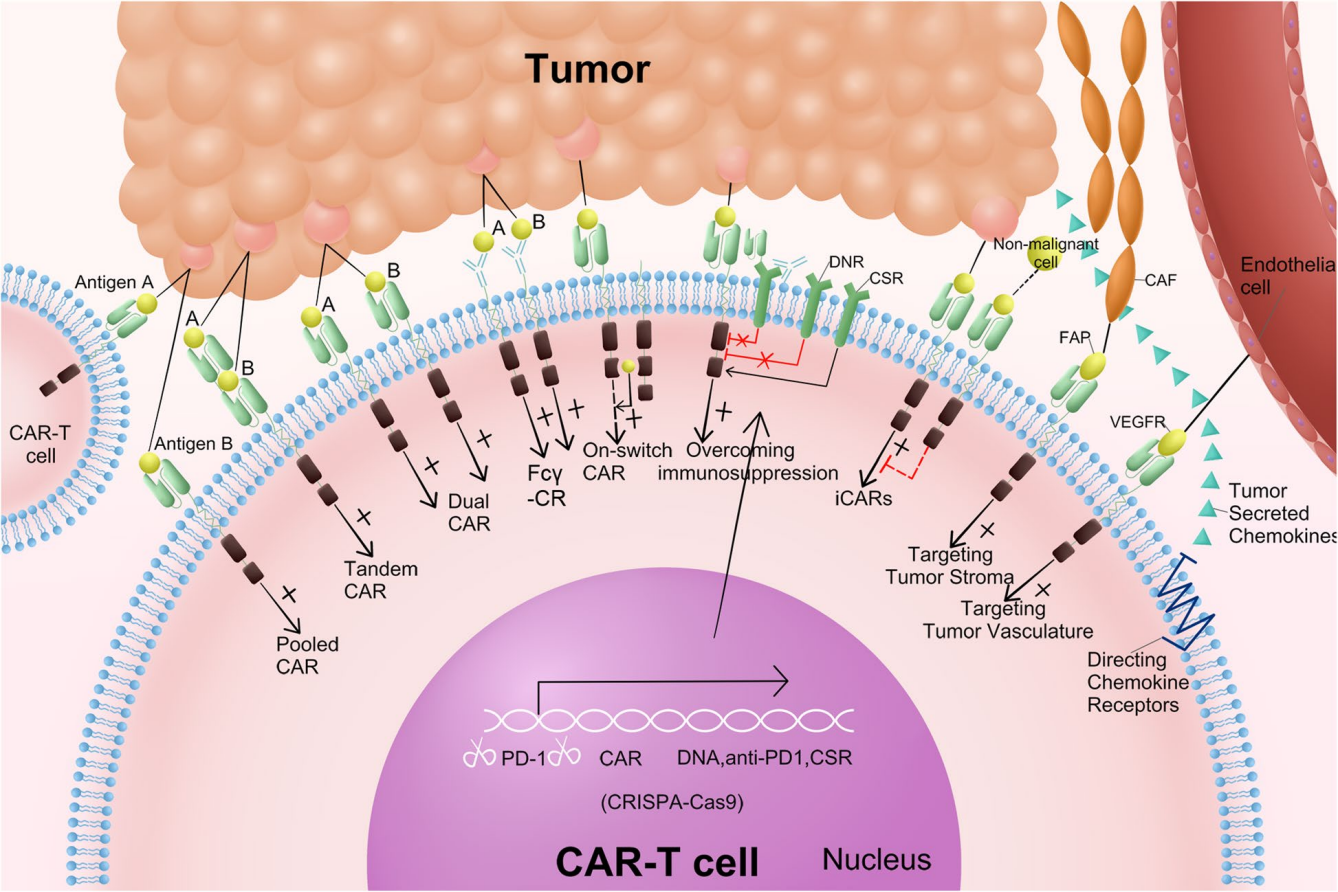
The strategies used to deal with the lack of ideal antigen or loss of antigen. iCAR: inhibitory CAR; DNR: dominant negative receptor; CSR: chimeric-switch receptor; FAP: fibroblast activation protein; VEGFR: vascular endothelial growth factor receptor; CAF: Cancer-associated fibroblast

Further efforts to identify the optimal therapeutic target are essential to refine CAR-T cell therapy. Many other strategies have been used to deal with the lack of ideal antigens or loss of antigens (Fig. 6). First of all, the implementation of targeting multiple antigens could compensate for the lack of target coverage and stability (Tables 2 and 3). T cells transduced by tandem CARs or multiple cis-transgenic CARs are multispecific CAR-T cells, representing the most widely used strategy today [444]. Fcγ-CR based on chimeric receptors composed of FcγR and T-cell signaling molecules and a continuous infusion of CAR-T cells targeting different targets could also achieve this goal [444]. Compared to classical CAR-T cells, combining Fcγ-CR T cells with specific mAbs allows for diagnosing and eliminating cancer cells by ADCC. The advantage of Fcγ-CR T cells is that the same Fcγ-CR T cells could target a variety of different TAAs, and the withdrawal of mAbs could reduce the release of cytokines [445]. Besides, CAR-T cells could circumvent inhibitory immune checkpoint activity by gene-editing techniques, such as rendering cells unable to recognize PD-L1 by CRISPR/Cas9 [446]. In addition, CAR-T cells could be modified to acknowledge tumor chemokines to overcome the difficulty of CAR-T cell transport to malignant tissue in solid tumor therapy, prolong the duration of themselves in cancer tissue and enhance the antitumor effect [444]. The specificity of CAR-T cells could be improved by either raising the expression of the target antigen in tumor tissue or finding the ideal target with minimal expression in healthy cells [447]. CAR-T cells could be used to attack not only tumor cells but also the solid tumor’s TME stroma and blood vessels.

**Table 2:** Multispecific CAR-T cell therapy in hematological tumors

Condition	Clinical trials (phase and number)	Targeting domain	Age	Enrollment	Start Date	Locations	Singaling domains	Response	Dose (cells/kg or cells/m2)	Toxicity	Reference
**B-NHL**	Phase 1 (NCT03233854)	CD19/CD22	Adult, Older Adult	21	1-Sep-17	Stanford University, School of Medicine; Palo Alto, California, United States	4-1BB-CD3ζ	6CR	3 × 10^6^	CRS gr. 3–4 (5%), Neurotoxicity gr.3–4 (0%)	[47]
	Phase 2 (NCT03196830)	CD19/CD22	Child, Adult, Older Adult	32	1-Jun-17	the First Affiliated Hospital of Soochow University the First Affiliated Hospital of Soochow University Suzhou, Jiangsu, China	4-1BB-CD3ζ	11CR	a total dose of 3.690× 10^8^–3.285× 10^9^	CRS gr. 3–4 (28%), Neurotoxicity gr.3–4 (12.5%)	[48]
	Phase 1 (ChiCTR1800015575)	CD19/CD22	Child, Adult, Older Adult	16	10-Apr-18	the First Affiliated Hospital, School of Medicine, Zhejiang University	4-1BB-CD3ζ	10CR	1–10× 10^6^	CRS gr. 3–4 (6%), Neurotoxicity gr.3–4 (0%)	[49]
	Phase 1/2 (NCT03097770)	CD19/CD20	Child, Adult, Older Adult	87	1-Apr-17	Biotherapeutic Department and Pediatrics Department of Chinese PLA General Hospital Beijing, Beijing, China	4-1BB-CD3ζ	61CR	0.5× 10^6^–8× 10^6^	CRS gr. 3–4 (10%), Neurotoxicity gr.3–4 (2%)	[50]
	Phase 1 (NCT03019055)	CD19/CD20	Adult, Older Adult	19	16-Oct-17	Froedtert Hospital & Medical College of Wisconsin, Milwaukee, Wisconsin, United States	4-1BB-CD3ζ	12CR	2.5 × 10^5^–2.5× 10^6^	unknown	[51]
		CD19/CD70,CD19/20/22,CD19/CD37									[421–423]
**B-ALL**	Phase 1 (ChiCTR1800015575)	CD19/CD22	Child, Adult, Older Adult	15	10-Apr-18	the First Affiliated Hospital, School of Medicine, Zhejiang University	4-1BB-CD3ζ	13CR	1.04× 10^6^–7.02× 10^6^	CRS gr. 3–4 (13%), Neurotoxicity gr.3–4 (0%)	[89]
	Phase 1 (NCT03233854)	CD19/CD22	Adult, Older Adult	17	1-Sep-17	Stanford University, School of Medicine, Palo Alto, California, United States	4-1BB-CD3ζ	15CR	3× 10^6^	CRS gr. 3–4 (6%), Neurotoxicity gr.3–4 (18%)	[47]
	Phase 1 (NCT03185494)	CD19/CD22	Child, Adult, Older Adult	6	1-Aug-17	Biotherapeutic Department and Pediatrics Department of Chinese PLA General Hospital Beijing, Beijing, China	4-1BB-CD3ζ	6CR	1.7× 10^6^–3× 10^6^	CRS gr. 3–4 (0%), Neurotoxicity gr.3–4 (0%)	[87]
	Phase 1 (NCT03289455)	CD19/CD22	Child, Adult	15	26-Jun-17	Great Ormond Street Hospital for Children NHS Foundation Trust London, United Kingdom; University College London Hospitals NHS Foundation Trust London, United Kingdom; Royal Manchester Children’s Hospital Manchester, United Kingdom	4-1BB-CD3ζ	13CR	0.3–2× 10^6^,3 × 10^6^,4.3–5× 10^6^	CRS gr. 3–4(0%), Neurotoxicity gr.3–4 (7%)	[424]
	Phase 1 (ChiCTR-ONC-17013648)	CD19/CD22	Child, Adult	21	4-Dec-17	Beijing Boren Hospital, Beijing, China	4-1BB-CD3ζ	14CR	0.486–5.0× 10^5^ (CD19), 0.32–5.0× 10^5^ (CD22)	CRS gr. 3–4 (0%), Neurotoxicity gr.3–4 (0%)	[86]
		CD20/CD22,CD19/CD123,CD19/BAFF-R									[82, 425, 426]
**T-ALL**		CD5/CD7									[66]
**AML**		CD123/CD19,FLT3/NKG2DL,CD123/CLL1,CD13/TIM3,CD123/FRβ									[427–431]
**CLL**	Phase 1 (NCT03019055)	CD19/CD20	Adult, Older Adult	3	16-Oct-17	Froedtert Hospital & Medical College of Wisconsin, Milwaukee, Wisconsin, United States	4-1BB-CD3ζ	2CR	2.5 × 10^5^–2.5× 10^6^	unknown	[51]
**MM**	Phase 1 (ChiCTR1800018143)	BCMA/CD38	Adult, Older Adult	23	1-Sep-18	Institute of Hematology, Union Hospital Affiliated to Tongji Medical College, Huazhong University of science and technology	4-1BB-CD3ζ	12CR	0.5,1.0,2.0,3.0 and 4.0× 10^6^	CRS gr. 3–4 (22%), Neurotoxicity gr.3–4 (0%)	[169]
	Phase 2 (ChiCTR1800017051)	BCMA/CD38	Adult, Older Adult	22	1-Jun-18	The first central hospital of Tianjin, Tianjin, China	4-1BB-CD3ζ	12CR	2× 10^6^ (BCMA), 2× 10^6^ (CD38)	CRS gr. 3–4 (27%), Neurotoxicity gr.3–4 (0%)	[170]
	Phase 2 (ChiCTR-OIC-17011272)	BCMA/CD19	Adult, Older Adult	62	1-May-17	The Affiliated Hospital of Xuzhou Medical University, Jiangsu, China	4-1BB-CD3ζ	37CR	1× 10^6^ (BCMA), 1× 10^6^ (CD19)	CRS gr. 3–4 (10%), Neurotoxicity gr.3–4 (3%)	[171]

**Table 3:** Multispecific CAR-T cell therapy in solid tumors

Condition	Targeting domain	Reference
HCC	CD70/B7H3	[189, 414, 432–434]
	GPC3/CD147	
	c-MET/PD-L1	
	GPC3/ASGR1 gp100/HER2	
Glioma	CD70/B7H3	[189, 374–376, 435, 436]
	HER2/IL13Rα2	
	EGFRvIII/IL13Rα2 OR EphA2; MOG/IL13Rα2 OR EphA2	
	EGFRvIII/IL13Rα2 OR CD133	
	HER2/IL13Rα2/EphA2	
	IL13Rα2/ EphA2	
GC	Trop2/PD-L1	[277]
PC	MSLN/CEA	[437, 438]
	MSLN/PSMA	
EC	CD70/B7H3	[189]
CRC	CD30/CEA; CD30/TAG70	[439]
BC	CD70/B7H3	[189, 414, 440–442]
	Her2/IGF1R	
	MUC1/HER2	
	ROR1/B7H3 gp100/HER2	
PCa	MSLN/PSMA	[437]
LCa	CD70/B7H3	[189, 204, 442]
	ROR1/B7H3 PSCA/MUC1	
OC	FLOR1/MSLN	[330, 333, 334]
	ALPPL2/MCAM ALPPL2/MSLN ALPPL2/HER2 PD-L1/MUC16	
MEL	CD70/B7H3 gp100/CSPG4 gp100/VEGFR-2	[189, 314, 443]

Furthermore, incorporating switches could control CAR-T cell activity to reduce the toxic side effects. For example, certain types of CAR-T cells, including synNotch, and inhibitory CAR (iCAR), could regulate cell activity through endogenous switches when recognizing specific antigens, thereby reducing extra-tumor tissue toxicity [448]. iCAR also uses dual antigen targeting similar to Tandem CAR, but iCAR could inhibit the activation of active CARs by binding to a second inhibitory receptor [449]. On-Switch CAR could conversely regulate the intensity and duration of CAR-T cell activity by exogenous administration, which is more controllable [450].

In addition to the effects of target selection, we mention other side effects and corresponding remedies here. As many T cells are activated in a short period, the release of cytokines increases explosively over a short period, leading to serious adverse effects such as CRS and macrophage activation syndrome [451]. Neurotoxicity is another profound side effect of CAR-T treatment, which is usually treated clinically with tolimumab (IL-6 receptor inhibitor) or glucocorticoids [451]. Due to the low number of lymphocytes in cancer patients, autologous CAR-T cells take longer to produce and are difficult to expand. Still, allogeneic CAR-T is subject to graft versus host disease (GVHD) and rejection reactions [452]. By knocking down endogenous T-cell receptors (TCR) and leukocyte antigen class I molecules (HLA), universal CAR-T cells could both reduce immune rejection during allogeneic transplantation and avoid immune attack by allogeneic T cells on the host organ (GVHD) [419]. And methods such as lymphadenectomy could also reduce the risk of rejection. In addition, immunoglobulin injections could be used to maintain immune function, and allogeneic HSCT could be used to ensure HSC function.

Multiple combinatorial strategies are available to enhance therapeutic efficacy whileusingwhile using CAR-T therapies [453]. For example, mRNA technology could improve the expression of hidden antigens in tumor cells and encode target-specific CAR [321]. Oncolytic virus (OV) regulates TME and influences the ability of the host to mount an anti-tumor immune response [454]. Moreover, stimulating and enhancing endogenous DC activity will maximize T cell engagement and activation [455]. Nanotechnology has also been used to improve CAR-T cell therapy in recent years. Patients could be screened prospectively for surface antigen expression to enhance the efficiency of subsequent clinical trials.

CAR-T will eventually become a tumor buster by continuously exploring suitable targets and optimizing design solutions. CAR-T cell therapy is believed to bring a bright future to cancer patients.

## Data Availability

Not applicable.

## Abbreviations

AML: acute myeloid leukemia
ANXA2: Annexin A2
ADCC: antibody-dependent cellular cytotoxicity
B-ALL: B-acute lymphoblastic leukemia
BAFF-R: B-cell activating factor receptor
BCMA: B-cell maturation antigen
BL: burkitt lymphoma
B-NHL: B-cell non-Hodgkin’s lymphoma
BC: breast cancer
CAR: chimeric antigen receptor
CAR-T cell: chimeric antigen receptor T cell
CTCAE: National Cancer Institute’s Common Terminology Criteria for Adverse Events
CBEs: cytosine base editors
CNS: central nervous system
CRS: cytokine release syndrome
CRR: complete remission rate
CR: complete remission
CSPG4: chondroitin sulfate proteoglycan 4
CLL1: C-type lectin like molecule 1
CLL: chronic lymphocytic leukemia
CAIX: carbonic anhydrase IX
ccRCC: clear cell renal cell carcinoma
CLDN: claudin
CEA: carcinoembryonic antigen
CLTX: chlorotoxin
c-MET: c-mesenchymal-epithelial transition factor
CSCs: cancer stem cells
CXCR: chemokine receptor
CRC: colorectal cancer
CDH6: cadherin 6
CC: cervical cancer
CDH17: cadherin 17
DIPG: diffuse intrinsic pontine glioma
DLBCL: diffuse large B-cell lymphoma
DMG: diffuse midline glioma
Dox: doxycycline
DLL3: delta-like ligand 3
DSBs: DNA double strand breaks
EFS: event-free survival
EGFR: epidermal growth factor receptor
EphA: Ephrin type-A receptor
EpCAM: epithelial cell adhesion molecule
EC: esophageal cancer
ESCC: esophageal squamous cell carcinoma
EAC: esophageal adenocarcinoma
EWS: Ewing’s sarcoma
FDA: U.S. Food and Drug Administration
FRβ: membrane-associated folate receptor β
FLT3: FMS-like tyrosine kinase 3
FL: follicular lymphoma
FAP: fibroblast activation protein
Fn14: factor-inducible 14
FRα: folate receptor α
GVHD: graft versus host disease
GPRC5D: G protein-coupled receptor class-C group-5 member-D
GD2: ganglioside2
GBM: glioblastoma
GPC3: glypican-3
GUCY2C: Guanylyl cyclase C
GC: gastric cancer
GS: γ-secretase
GSI: γ-secretase inhibitor
HSC: hematopoietic stem cells
HL: Hodgkin’s lymphoma
HNSCC: head and neck squamous cell carcinoma
HER2: human epidermal growth factor receptor 2
HCC: hepatocellular carcinoma
HSCT: hematopoietic stem cell transplantation
ICAM-1: intercellular adhesion molecule-1
IGF1R: type I insulin-like growth factor receptor
iCAR: inhibitory CAR
LILRB4: leukocyte immunoglobulin-like receptor-B4
LCa: lung cancer
MHC: major histocompatibility complex
MSLN: mesothelin
MPL: myeloproliferative leukemia protein
mPFS: median progression-free survival
MM: multiple myeloma
MUC1: mucin 1
MEL: melanoma
NK cells: natural killer cells
NHL: non-Hodgkin’s lymphoma
NKG2D: natural killer group 2 member D
NY-ESO-1: New York esophageal squamous cell carcinoma-1
NSCLC: non-small cell lung cancer
NKG2DL: natural killer group 2 member D ligand
OC: ovarian cancer
OS: osteosarcoma
OR2H1: olfactory receptor 2H1
OR: objective response
ORR: objective response rate
PD-1: programmed cell death 1
PCNSL: primary central nervous system lymphoma
PODXL: Podocalyxin
PDPN: Podoplanin
PR: partial remission
PSMA: prostate-specific membrane antigen
PCa: prostate cancer
PSCA: prostate stem cell antigen
PAK4: p21-activated kinase 4
PD-L1: programmed death-ligand 1
PC: pancreatic cancer
PLAP: placental alkaline phosphatase
PTK7: protein tyrosine kinase 7
PFS: progression-free survival
r/r B-ALL: relapsed or refractory B-acute lymphoblastic leukemia
ROR: receptor tyrosine kinase like orphan receptor
r/r MM: relapsed or refractory multiple myeloma
RTK: receptor tyrosine kinase
RCC: renal cell carcinoma
scFv: single-chain fragment variable
SLAMF7: signaling lymphocytic activation molecule F7
SLAMF3: signaling lymphocytic activation molecule F3
SCLC: small cell lung cancer
TAA: tumor-associated antigens
TCR: T cell receptor
T-ALL: T-acute lymphoblastic leukemia
TSLPR: thymic stromal lymphopoietin receptor
TIM-3: T cell immunoglobulin and mucin structural domain 3
T-NHL: T-cell non-Hodgkin’s lymphoma
TRBC: T cell receptor β-chain constant domains
TME: tumor microenvironment
TGFβ: transforming growth factor-β
TROP2: trophoblast cell surface antigen 2
TC: thyroid cancer
VEGFR-2: vascular endothelial growth factor receptor 2
VHH: single variable domain on a heavy chain
WT1: wilms tumor 1
